# Higher-Level Strategies for Computer-Aided Retrosynthesis

**DOI:** 10.1021/acscentsci.5c02014

**Published:** 2026-03-05

**Authors:** Jihye Roh, Joonyoung F. Joung, Kevin Yu, Zhengkai Tu, G. Logan Bartholomew, Omar A. Santiago-Reyes, Mun Hong Fong, Richmond Sarpong, Sarah E. Reisman, Connor W. Coley

**Affiliations:** † Department of Chemical Engineering, 2167 Massachusetts Institute of Technology, Cambridge, Massachusetts 02139, United States; ¶ Center for Computational Science and Engineering, Massachusetts Institute of Technology, Cambridge, Massachusetts 02139, United States; § Department of Electrical Engineering and Computer Science, Massachusetts Institute of Technology, Cambridge, Massachusetts 02139, United States; ∥ Department of Chemistry, 1438 University of California, Berkeley, Berkeley, California 94720, United States; # Division of Chemistry and Chemical Engineering, 6469 California Institute of Technology, Pasadena, California 91125, United States

## Abstract

Retrosynthesis is a core technique in organic chemistry that simplifies target molecules into more readily available components. Computer-aided synthesis planning (CASP) automates this process by recursively proposing immediate precursors to identify multistep synthetic pathways. However, CASP typically struggles for complex molecules that require longer synthetic pathways and present a greater number of possible disconnections. Here, we introduce a new *higher-level* framework for computer-aided retrosynthesis. Our approach abstracts detailed substructures in pathway intermediates not appearing in the target product, allowing the algorithm to emphasize higher-level strategies while postponing the consideration of specific functional group choices, thus reducing the effective width and depth of the search space. This framework achieves higher top-*k* accuracy in single-step retrosynthesis and identifies multistep routes for more targets than the original approach. Through case studies on complex drugs and natural products, we demonstrate how routes proposed by our framework provide a powerful basis for developing full synthesis plans, particularly in challenging cases where the original approach fails, while enabling chemists to leverage their expertise to refine the synthesis design. Ultimately, focusing on higher-level strategies enables an effective and intuitive approach for challenging targets in computer-aided retrosynthesis.

## Graphical abstract

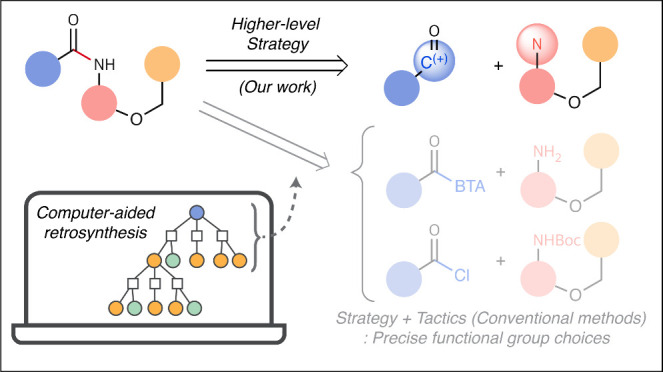

## Introduction

Organic synthesis enables chemists to synthesize molecules that are scarce in nature, as well as their synthetic analogs, and underpins many applications including the development of small molecule therapeutics. To plan synthetic pathways, chemists have long utilized retrosynthetic analysis to progressively break down the target molecule into simpler, more accessible precursors. Despite this systematic planning approach, synthesizing complex molecules requires extensive exploration of a vast space of hypothetical routes, which can be challenging even for experienced chemists.

Computer-aided synthesis planning (CASP), initially formulated by E. J. Corey in the 1960s,^1^ aims to assist chemists in their efforts by automating the process of retrosynthetic analysis. A typical CASP algorithm recursively proposes precursors for molecules along a synthetic route, searching for multistep syntheses that decompose complex molecules into readily available building blocks^2–10^ (Figure 1A). While CASP tools are effective for many targets, synthesis planning of complex molecules,^11^ both synthetically and structurally, presents significant challenges,^12^ especially due to the inherent need for longer synthetic routes and the greater number of potential disconnections that might be feasible at each step. These challenges exacerbate the combinatorial explosion of potential pathways to consider in the multistep search. Assessing the feasibility of routes after ideation in a robust, automated manner, and including considerations such as yields and side products, is also highly challenging, though beyond the scope of this work.

**Figure 1:**
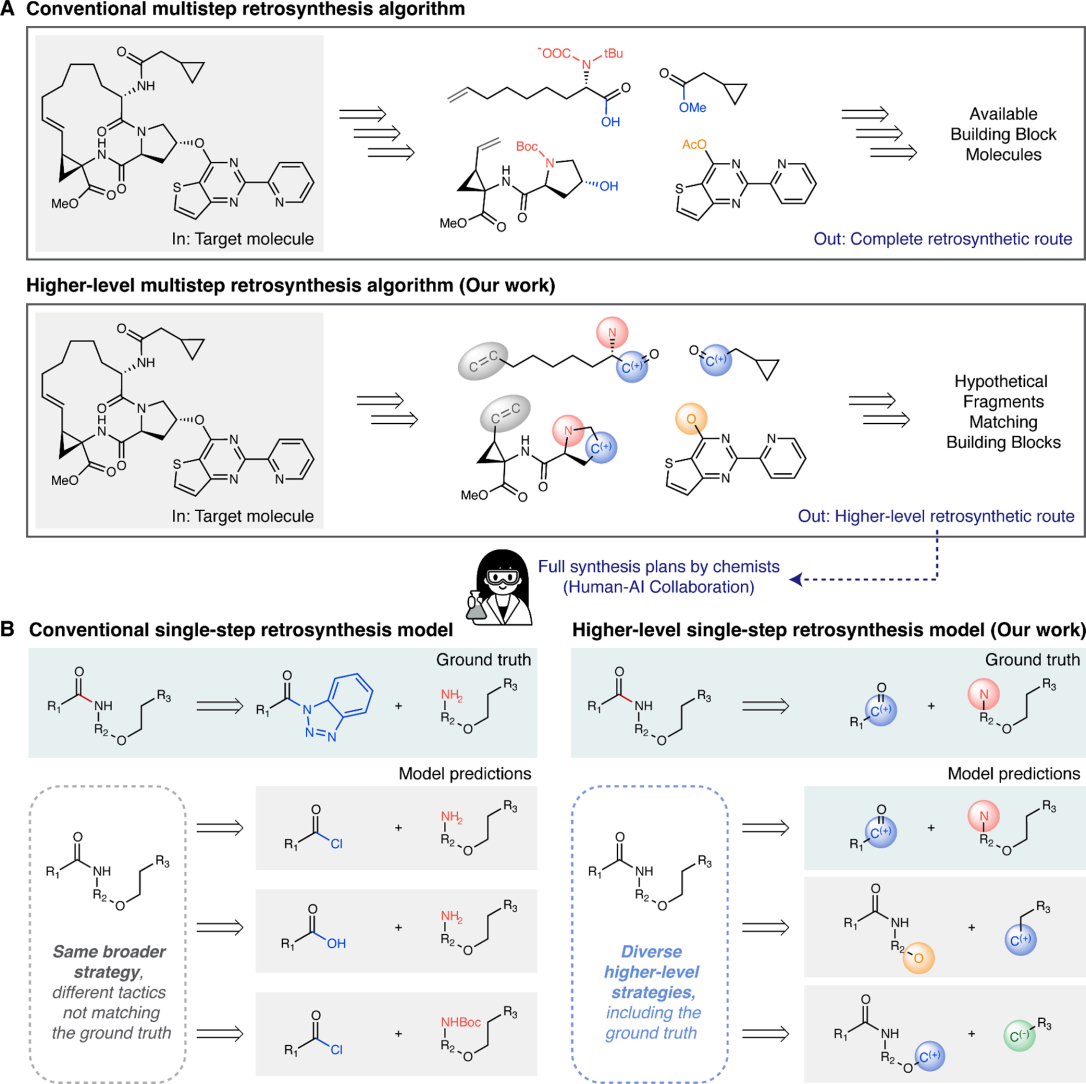
Overview of conventional and higher-level retrosynthesis algorithms. (A) Comparison of conventional (top) and higher-level (bottom) multistep retrosynthesis algorithms. Conventional methods involve planning each reaction in detail, including protecting group manipulations, functional group interconversions, and choice of leaving groups. Our higher-level approach abstracts the leaving substructures, resulting in *synthon*-like structures, and instead focuses on scaffold-building steps. The routes proposed by our higher-level algorithm can subsequently be translated into full synthesis plans by chemists, enabling effective human–AI collaboration in synthesis planning. (B) Comparison of conventional (left) and higher-level (right) single-step retrosynthesis models used in multistep algorithms. Conventional models propose precursors that involve various leaving groups for the same general strategy. By abstracting the specific functional groups, our single-step model focuses on the overall retrosynthetic strategy and effectively consolidates chemically similar proposals into one representation, matching the ground truth while proposing more diverse strategies.

Many approaches to computer-aided retrosynthesis have been explored. Early CASP tools used expert-encoded reaction rules and heuristics to propose and select transformations.^2–5,13–16^ A notable advancement in this area is Synthia (formerly known as Chematica),^7,17^ which uses highly curated expert reaction rules and heuristics to promote chemically sound, strategic disconnections and ensure that the branching factor (i.e., the number of proposed reactions for a given intermediate) remains low. By leveraging increased computational power and advancements in network algorithms in recent years, Synthia has generated plausible synthetic pathways for diverse targets, including those for natural products that were evaluated as equivalent in quality to published routes in a blinded study, with several designs validated in the laboratory.^18,19^

In parallel, data-driven alternatives have been developed that leverage chemical knowledge from large reaction data sets and recent advancements in machine learning to propose retrosynthetic transformations. Single-step retrosynthesis models propose immediate precursors (i.e., reactants) for target products, offering scalability to large molecular spaces and enabling the exploration of a broad range of transformations.^8–10^ In addition to proposing viable single-step precursors, significant efforts have been made to better guide the navigation toward viable multistep synthetic pathways. Various search algorithms are employed to automatically select the next intermediate to propose precursors for, often prioritizing those more likely to lead to commercially available building blocks.^19–23^ Furthermore, recent algorithms have increasingly incorporated user input, allowing chemists to specify one or more starting materials^24–27^ or indicate specific bonds to break/maintain,^28^ thus tailoring the exploration of synthetic pathways to specific needs.

However, modern computational approaches do not align well with the way a chemist approaches synthesis planning. Many chemists will start by identifying high-level disconnections that break molecules into *synthons* (i.e., generalized, hypothetical fragments).^29–32^ This method allows chemists to focus on the broader retrosynthetic *strategy* first before addressing *tactics*–the specific conditions and functional groups necessary to achieve the desired transformation in a retrosynthetic pathway,^33–36^ enabling the selection and modification of these details based on feasibility, yield, or downstream compatibility (Supporting Information Section S2). In contrast, existing computational approaches to multistep retrosynthesis generally consider both strategy and tactics together at the single-step level. By specifying all functional groups in their suggestions, they often propose reactions with the same disconnections but different functional groups, as well as tactical reactions such as functional group interconversions (FGIs) (Figure 1). While efforts have been made to consider synthons or synthetic strategy in synthesis planning, existing methods either operate at the single-step level, where synthons are identified but subsequently completed with functional groups (i.e., semitemplate-based methods),^37–44^ or at the evaluation stage, assessing the strategy of the pathways after they have been generated.^45,46^

Thus far, a multistep retrosynthetic algorithm that simultaneously addresses strategy and tactics with data-driven models, regardless of whether the underlying single-step model performs synthon-like reasoning, has been insufficient in overcoming the challenges of planning syntheses for complex molecules. In this work, we propose a framework that focuses on the broader retrosynthetic strategy, significantly reducing the combinatorial complexity of the problem and aligning more closely with how expert chemists conceptualize retrosynthesis. Our *higher-level* retrosynthetic planning algorithm emphasizes scaffold-building reactions and proposes multistep pathways of *synthon*-like structures corresponding to sets of molecules, avoiding specifying leaving groups or protecting groups upfront (Figure 1). In this framework, experimental multistep pathways are systematically abstracted into higher-level pathways that capture recurring strategic patterns observed in the literature. For example, at this higher-level, we treat carboxylic acids, esters, and acid halides as the same acyl cation equivalent, regardless of the leaving group, and exclude transformations that merely interconvert these functional group variants in order to focus on the underlying synthetic strategy. Such abstraction of functional groups is not intended to disregard functional group effects, but rather to acknowledge that, for each abstracted transformation, there typically exists at least one instantiation of functional groups that can implement the strategy. Functional group assignment and optimization are deferred to later stages, where chemical context and feasibility can be more effectively assessed.

We curate a new data set of such higher-level reactions (i.e., reactions with detailed leaving substructures abstracted) instead of complete reactions. We leverage this data set to train a data-driven single-step retrosynthesis model that learns higher-level strategies embedded in large-scale reaction data to propose plausible strategies for target molecules beyond those in the reaction data set (Figure 1B). We develop a multistep synthesis planning algorithm that operates at this higher-level, which demonstrates significant improvements in its ability to efficiently navigate the combinatorial space of hypothetical pathways by prioritizing core transformations and consolidating proposals with equivalent synthetic strategies. In addition, we illustrate how the proposed higher-level strategies can be translated into complete synthesis plans by chemists, allowing the selection of specific functional groups with the context of the full pathway in mind. Ultimately, our higher-level framework provides an effective, chemist-aligned strategy for computer-aided retrosynthesis planning.

## Results and Discussion

### Higher-Level Reaction Data Set Curation

We used the publicly available USPTO-Full data set^47^ with ∼1.8 M reactions to generate the higher-level reaction data set (Figure 2A). After processing the reactions, we removed the reactions sharing the same reactant-product pairs as those in the USPTO-190 data set,^23^ a set of 190 target molecules with synthetic routes extracted from the reactions in the USPTO-Full data set. We later use these target molecules to evaluate the performance of our algorithm. This resulted in a data set of ∼1.4 M single-product reactions (802,024 unique reactant-product pairs), hereafter called the original reaction data set. See Section S3 in Supporting Information for more details.

**Figure 2:**
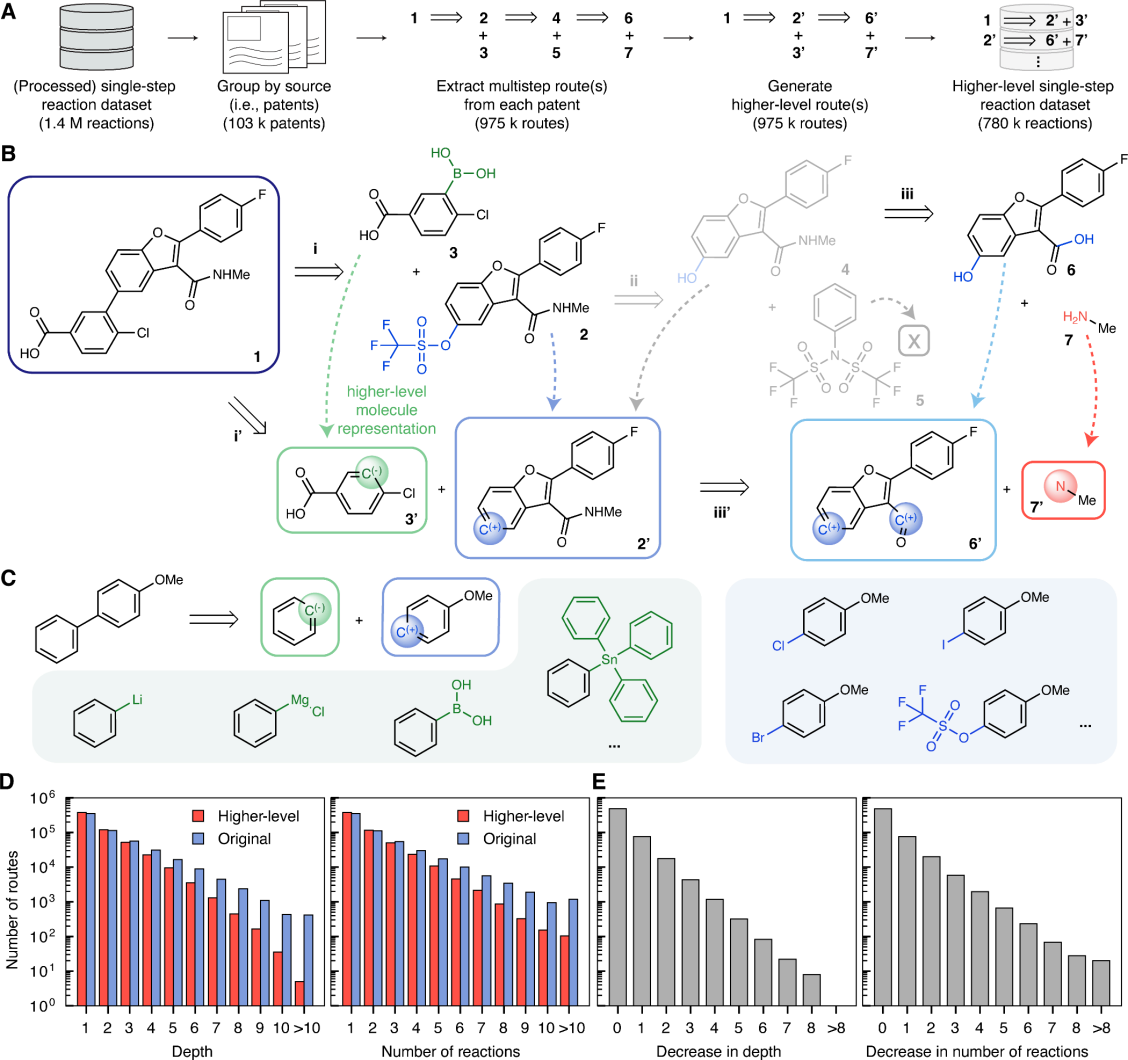
Curation of higher-level route and reaction data sets. (A) Overview of data set curation process. (B) Example extracted pathway consisting of three reactions (top) and the corresponding higher-level route with two steps (bottom). Atoms in the pathway not appearing in the final product are automatically identified and abstracted, forming the higher-level representation with the abstracted group visualized in spheres. Molecules and reactions removed in this process are shown in gray. The hydroxyl to triflate interconversion ii, which enables the subsequent Suzuki coupling i, is removed, resulting in a two-step higher-level route. (C) Example molecules corresponding to each higher-level molecule. Distinct molecules in the original space are consolidated into one representation in the higher-level space. (D) Distributions of depth (left) and the number of reactions (right) for higher-level (red) and original (blue) routes. (E) With abstraction, the depth decreases by 0.223 steps (left) and the number of reactions decreases by 0.248 (right) per route on average.

Multistep routes were extracted following the workflow of Mo et al.,^45^ which constructs and traverses a network of single-step reactions to identify retrosynthetic pathways in each patent. The atoms in the target molecule were traced back to the starting materials to identify the leaving atoms (i.e., those that do not appear in the target molecule) along the pathway. Then, higher-level routes were generated by removing the identities of these leaving atoms, resulting in *synthon*-like higher-level representations for each molecule, with the nonleaving (i.e., core) atom connected to the leaving structure marked to indicate it being an abstracted “group” (Figure 2B).

Specifically, our abstraction heuristics are based on leaving groups’ electron affinities and the identities of heteroatoms. That is, abstracted groups with a heteroatom as the core atom are represented as a single entity, while those with carbon atom as the core atom are subdivided based on the electronegativity of the leaving structure. For example, any chloro-, bromo-, iodo-, or triflyl-type Suzuki, Stille, or Kumada coupling is abstracted as a single-step C–C coupling between an electrophilic C^(+)^ group and a nucleophilic C^(−)^ group (Figure 2C). Note that the symbols (+) and (−) indicate the relative electronegativity of the carbon atom compared to the connected atom in the leaving group, rather than a formal charge, inspired by the D. A. Evans formalism of charge affinity patterns.^31^

This abstraction process leads to a higher-level representation of molecules, where different specific functional groups are abstracted into the same form. As a result, distinct reactions in the original data set are consolidated into the same reaction in the higher-level data set. Moreover, this abstraction process removes reactions that solely affect the leaving substructures, such as FGIs of leaving functional groups, leading to higher-level routes that focus on transformations necessary to build the target product (Figure 2B). Consequently, the average depth (i.e., number of reactions in the longest linear sequence) and number of reactions decrease in the higher-level routes compared to the original routes they were generated from (Figure 2D, E).

Our final higher-level data set consists of each reaction in the higher-level routes, containing 780,115 unique reactions (i.e., reactant-product pairs) in total. Additional details on our abstraction heuristics and examples of how nonpolar couplings, cycloadditions, and other relevant transformations are handled by our method are provided in Section S4 of the Supporting Information. Statistics on the abstracted groups and representative examples are provided in Supporting Information Section S4.5.

Importantly, because our abstraction heuristics are based primarily on atomic identity and electronegativity rather than predefined specific leaving groups or substructures, they can be readily applied to other reaction data sets, including those with newly developed transformations. To demonstrate the generality of our heuristics beyond the USPTO-Full data set, we applied the same method to the larger, more diverse Pistachio data set.^48^ Results with the Pistachio data set can be found in Supporting Information Section S9.

### Higher-Level Single-Step Retrosynthesis Model

For the single-step retrosynthesis model, we opted for a template-based approach that aims to predict the most chemically sound reaction templates for a given molecule. These templates represent general reaction rules for transformations in submolecular patterns and provide inherent explainability, as each template can be traced back to the literature precedents it was extracted from. They define the space of transformations considered by the model based on retrosynthetic strategies directly inferred from experimentally reported chemistry. Importantly, while demonstrated here with a specific template-based model, our framework is flexible and can be extended to any single-step model architecture, including other template-based approaches^49,50^ as well as template-free models.

We trained two single-step models: one with the higher-level data set and another with the original data set for comparison (hereafter referred to as the higher-level and original single-step models, respectively). The reactions in each data set were deduplicated based on reactant-product pairs, and reaction templates were automatically extracted from each reaction using a modified version of RDChiral^51^ capable of handling abstracted group representations. Templates were consolidated by applying all extracted templates to each reaction’s product and identifying the most general template able to recover the recorded reactant(s). This procedure yielded 32,622 and 51,736 templates for the higher-level and original data sets, respectively. The three templates with the greatest number of reaction precedents are shown in Figure 3A. Template consolidation, conceptually similar to generalization of templates by Chen and Jung^49,52^ and consistent with their reported benefits, improved both single-step model accuracy (Figure S8) and multistep success rates (Table S5). See Sections S5.1.1 and S5.1.2 in Supporting Information for more details.

**Figure 3:**
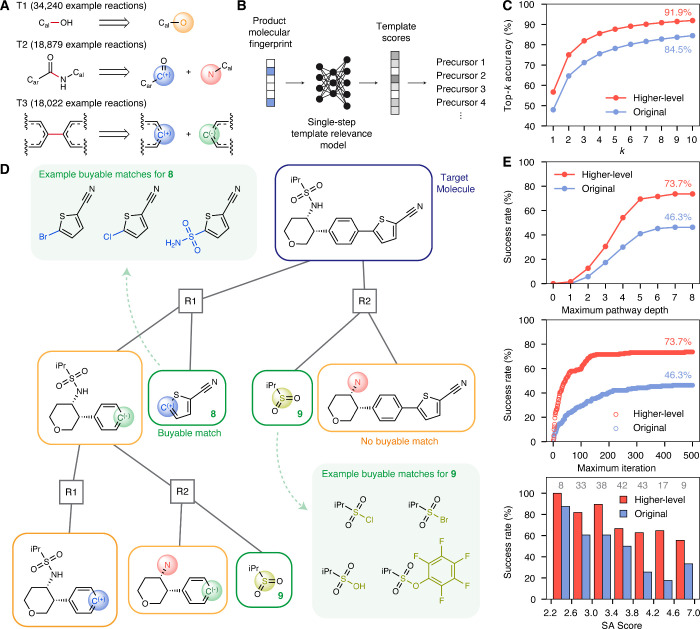
Schematic and performance of the higher-level retrosynthesis algorithm. (A) Three templates (T1–T3) from the higher-level data set with the greatest number of reaction precedents. T1 encodes an alcohol deprotection or ether cleavage; T2 encodes an amide coupling between an aryl-adjacent acyl cation equivalent and (protected) alkyl amines; and T3 encodes a biaryl cross-coupling between nucleophilic and electrophilic carbons. (B) Workflow of the template-based single-step model. Given the product fingerprint, the model predicts template scores, and the top ranked templates are applied to the product to generate precursors. (C) Single-step accuracies for the higher-level (red) and original (blue) models. (D) Example multistep search tree showing two iterations with two selected reactions per iteration. (E) Multistep planning success rates for higher-level (red) and original (blue) algorithms for the USPTO-190 molecules as a function of maximum depth of routes (top), maximum number of iterations (middle), and SA Score of the target molecule (bottom). The number of molecules in each binned SA Score interval are indicated in gray. The last bin includes all molecules with SA Score ⩾ 4.6.

The deduplicated reactions in each data set were randomly split 80/10/10 into training/validation/test sets. The template relevance module from ASKCOS^53^ was used to train the single-step retrosynthesis model following the approach of Segler et al.^54^ Given a product molecular fingerprint, a simple feedforward neural network is trained to suggest likely templates as a classification task, and the templates are applied to the product molecule to generate precursors (Figure 3B). Model hyperparameters were selected based on performance on the validation set. More details are provided in Section S5.1.3 in Supporting Information.

Single-step model performance was evaluated in terms of the top-*k* accuracy, which measures the percentage of products where the ground truth (i.e., literature-recorded) precursor is in the highest-ranked *k* predictions by the model. Since a template may give multiple precursors, we adopt the pessimistic definition of top-*k* accuracy, where the ground truth precursor, if present, is ranked last among the set of precursors generated by the same template. The high top-*k* accuracy in the higher-level model illustrates its ability to learn the general synthetic strategy, with 91.9% of the ground truth abstracted precursors (i.e., strategies) being captured within the top 10 predictions (Figure 3C, Section S5.1.4 in Supporting Information). While the higher-level and original models address different tasks and are not directly comparable in a quantitative sense, Figure S9 offers a qualitative example showing that the higher-level model proposes a more diverse set of retrosynthetic transformations, and that the top-*k* accuracy in the higher-level space better reflects alignment with broader literature strategies, which may not always be captured by exact-match metrics in the original space.

### Higher-Level Multistep Retrosynthetic Planning

The higher-level multistep retrosynthetic planning algorithm is built on the Monte Carlo tree search algorithm implemented in ASKCOS (referred to here as the “original” algorithm for comparison).^20,53^ In both higher-level and original algorithms, the multistep search is initiated with the input target molecule as the root node of the search graph, followed by an iterative process involving a select → expand → update sequence until some stopping criterion is met. At the selection step, an unexpanded, nonterminal (i.e., nonbuyable) chemical node is selected. At the expansion step, the corresponding single-step model is used to propose precursors for the selected molecule. Then, the original algorithm checks whether the exact species in each of the newly added molecule nodes is in the list of buyables. The higher-level algorithm uses substructure search to find buyable molecules that structurally match the newly added nodes (Figure 3D). The scores for each relevant node are then updated to reflect the newly added nodes. Once the stopping criterion (e.g., maximum number of iterations) is met, the explored network is traversed to identify routes from the root node (i.e., target product) to the terminal nodes. In the higher-level algorithm, the matched buyable molecules for the terminal nodes serve as a guide for users when selecting specific building blocks for forward synthesis. These buyable matches can be further sorted based on various user-defined criteria, such as feasibility, as a postprocessing step. See Section S5.2 in Supporting Information for more details on the algorithm, and Section S7 for the feasibility analysis and ranking of buyable molecules.

To evaluate the performance of our algorithm, we performed an automated multistep retrosynthesis search using both the higher-level and original retrosynthetic planning algorithms with the target molecules in the USPTO-190 data set (see Section S6 in Supporting Information for more details). The quantitative performance of our algorithm is evaluated in terms of success rate, defined as the percentage of target molecules for which the algorithm could identify at least one route that terminates in the specified buyables or their structural matches. The results demonstrate that the higher-level algorithm proposes routes for a greater number of molecules within the same depth and iteration (i.e., single-step model call) limit, including more complex molecules as measured by commonly used metrics of synthetic and structural complexity such as Synthetic Accessibility (SA) Score^55^ (Figures 3E and S10, Table S5).

We further analyze the differences in routes successfully identified by the two algorithms for the same target in order to demonstrate the mechanisms by which the higher-level formulation improves multistep planning performance. By generalizing at the level of strategy, the higher-level algorithm can draw wider analogies with precedents to be more creative when beneficial. For example, the cyclization of **10**, the higher-level equivalent of **14**, represents a general cyclization strategy involving a C^(+)^ group, which can be extended to various functional groups such as acetal groups or hydroxyl groups (Figure 4A). Using such generalization, the higher-level algorithm is able to propose disconnections following the same general strategy as the original algorithm, while bypassing tactical reactions such as redox manipulations. As a consequence of removing these tactical steps during planning, the final routes proposed by the higher-level algorithm exhibit reduced depth and fewer reactions compared to those proposed by the original algorithm (Figure 4B, Table S5).

**Figure 4:**
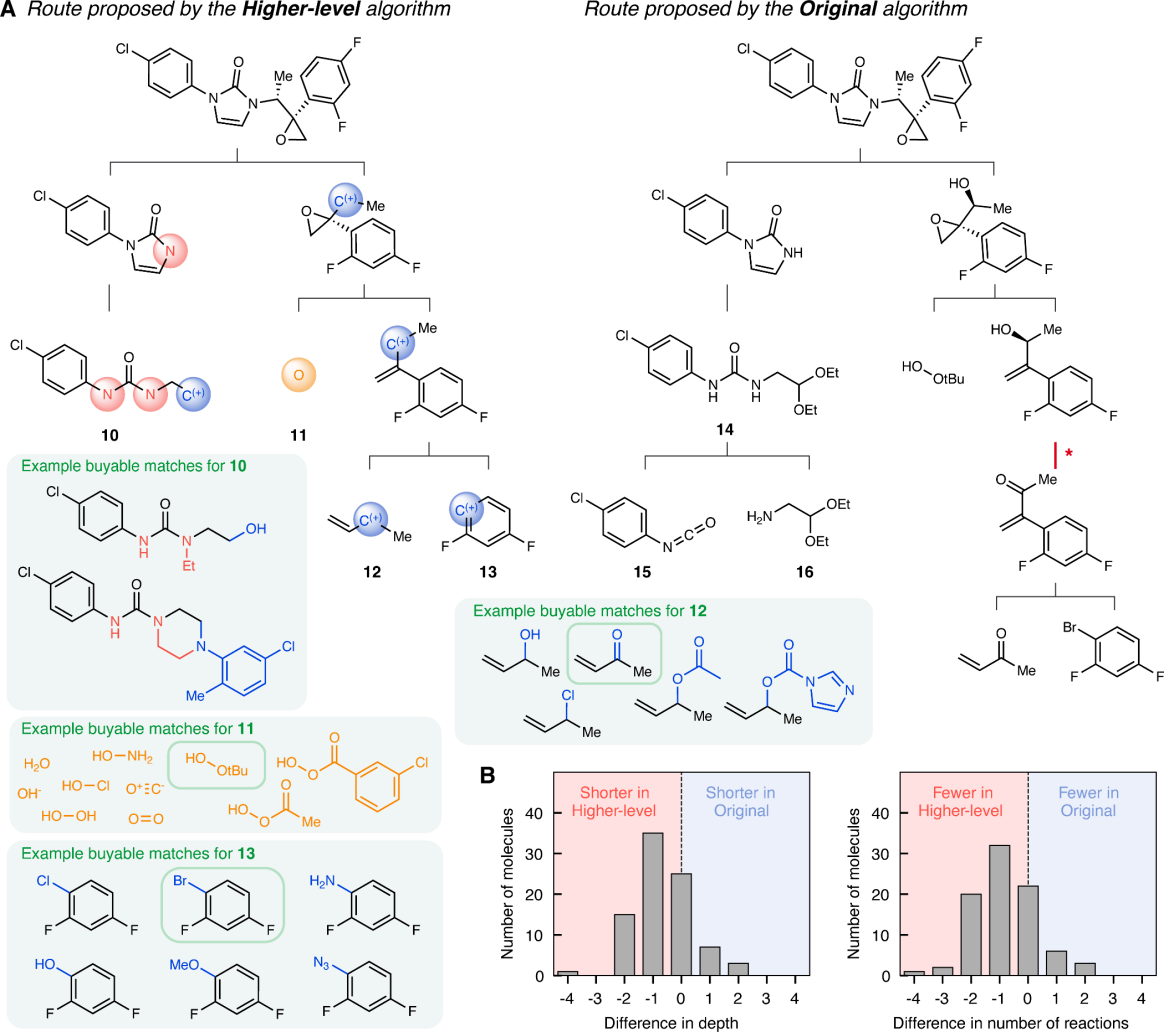
Comparison of routes successfully identified by both the higher-level and original algorithms for USPTO-190 molecules. (A) Example routes proposed by the higher-level (left) and original (right) algorithms, selected to illustrate when both algorithms yield equivalent strategies for the same target molecule. The higher-level algorithm identifies the same strategy while forgoing reactions like * (red), proposing a shorter pathway (depth three, four reactions) compared to the original algorithm (depth four, six reactions). Examples of buyable molecules matching each of the starting materials in the higher-level route are displayed in green boxes, and the exact starting materials used in the original route are shown in green outlines. (B) Difference in the depth (left) and the number of reactions (right) in the routes proposed for the same target molecules. The difference is calculated with the shortest depth and fewest number of reactions for each target which both algorithms successfully proposed routes for (86 molecules). The routes proposed by the higher-level algorithm are shorter by 0.65 and have 0.81 fewer reactions per molecule on average.

The exclusion of tactical reactions at this stage does not indicate that they are unnecessary in a fully specified synthesis; rather, their consideration is postponed so that the algorithm operates over a reduced abstract search space. The resulting reduction in route depth reflects properties of the planning representation rather than fewer experimental steps, directly lowering search complexity and enabling more efficient use of computational resources. This, in turn, allows the algorithm to identify retrosynthetic strategies beyond those proposed by the original approach, as demonstrated by its ability to identify routes to targets for which the original algorithm cannot find any.

### Case Study: Narlaprevir

To further illustrate how the algorithm performs in practical settings, we conducted multistep pathway searches using drugs and natural products with known synthesis pathways as targets^34,56^ (See Section S8 in Supporting Information for details). A notable example demonstrating our algorithm’s effectiveness is narlaprevir, an oral drug developed for the treatment of chronic hepatitis C that inhibits the NS3/4A serine protease.^57^ The automatic multistep search with our higher-level algorithm was able to identify routes for narlaprevir at iteration 25, while the original algorithm required 331 iterations to propose the first route.

One of the routes proposed by our algorithm is shown in Figure 5A, which closely matches the disconnections in the synthesis of narlaprevir in literature (Figure 5B).^58^ The proposed retrosynthesis begins with a C–N bond-formation between α-ketoamine **18** and proline derivative **25**. In a forward sense, this disconnection is likely accomplished by a peptide coupling. From there, a similar C–N bond formation is used to install the cyclopropylamine fragment of α-ketoamine **18** from α-ketocarbonyl **20**. An enantioselective oxidative α-amination could be used to forge the α-aminoketone of **20** from **22**, which could arise from N-butylation of α-ketocarbonyl **24**. For the other peptide coupling partner – proline derivative **25** – the endocyclic nitrogen atom of **26** and the pendant carbonyl of urea **27** are linked by an additional peptide coupling reaction. Urea **27** arises from addition of α-aminocarbonyl **28** into the isocyanate moiety of sulfone **29**, which itself could arise from a substitution and oxidation sequence from buyable matches of cyclohexylcarbonyl **33**.

**Figure 5:**
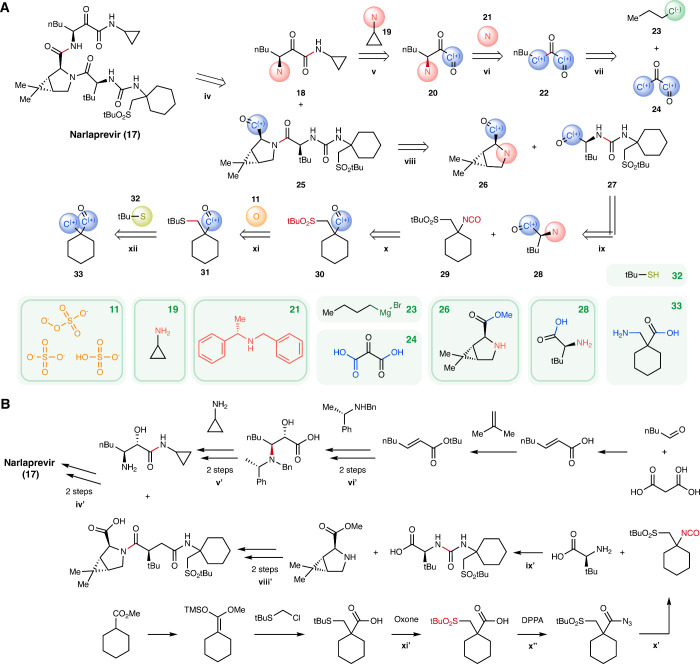
Case study: Narlaprevir. (A) A route proposed by the higher-level algorithm for the drug narlaprevir, with the disconnected and/or changed bond shown in red. (B) The literature route for narlaprevir.^58^ Seven out of the nine steps shown in the proposed higher-level route are transformations used in the synthesis of narlaprevir in literature (iv–vi, viii–xi). The reactions in the reference route corresponding to *r*_*i*_ in the higher-level route are labeled *r*_*i*_^′^ or *r*_*i*_^″^. The disconnected bonds in these steps are highlighted in red. Examples of one buyable molecule matching each of the starting materials are displayed in green boxes, and the exact starting materials used in the literature are highlighted (green outline). The higher-level algorithm is able to identify five starting materials used in the literature route. Reactions in the literature route, such as the deprotection of an amine, were ignored in this route, as they do not build up the core scaffold of narlaprevir.

Note that the last two steps, **vii** and **xii**, are different from the disconnections used in the literature; the literature uses a Doebner modification of the Knoevenagel condensation with malonic acid and pentanal instead of **vii**, and a reaction of silyl enol ether with a chloro-substituted sulfide to produce an ester instead of **xii** in the proposed route. While our algorithm is not able to exactly recover the literature disconnections with the current search parameters, our algorithm proposes alternative disconnections and identifies buyable molecules that match the starting molecules, resulting in a successful identification of a sequence of transformations that can be used to synthesize the drug. Thus, our algorithm is able to suggest a synthetic route closely resembling the reference pathway and also provides alternative starting materials and reactions, offering chemists flexibility to customize their synthesis according to specific needs.

### Case Study: Pinolidoxin

An additional example where the higher-level algorithm demonstrated its effectiveness is in identifying synthetic routes for (+)-pinolidoxin, a phytotoxic nonenolide natural product isolated from fungus *Ascochyta pinodes*,^59^ and its enantiomer (−)-pinolidoxin.^60^ While the original algorithm was unable to propose any synthetic routes for either enantiomer, the higher-level algorithm successfully proposed routes for both enantiomers.

The shortest route identified by the higher-level algorithm was for (−)-pinolidoxin (**34**). In this shortest route (Figure 6A), the algorithm proposes a reductive coupling between two polarity-matched carbon atoms to forge the 10-membered oxacanone ring of the pinolidoxin (**34**) scaffold. The preceding ring-opened ester **35** is formed through a C–O bond formation between glycol **36** and dienoate **39**. Vicinal diol **36** is proposed to arise from a diastereoselective C–C bond-forming reaction between carbon nucleophile **37** and an electrophilic carbon atom on buyable glycol **38**. This could be achieved by an asymmetric organometallic addition of an *N*-propyl nucleophile. The corresponding dienoate fragment, **39**, may be formed by an umpolung-type α-functionalization of carboxylate **40**, which corresponds to several buyable molecules, with buyable dienoic acid **41**.

**Figure 6:**
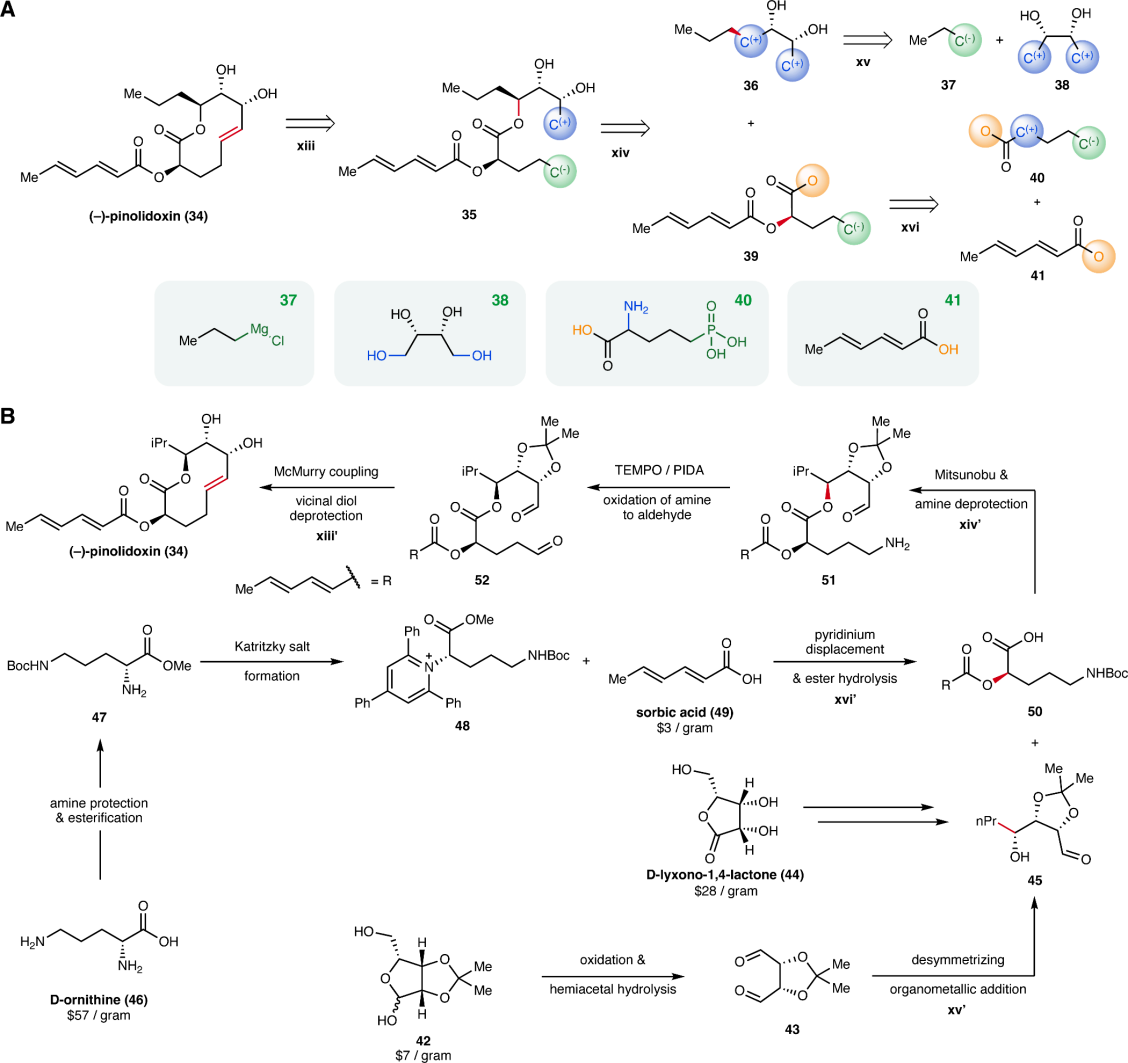
Case study: Pinolidoxin. (A) The shortest route proposed by the higher-level algorithm for (−)-pinolidoxin, with the disconnected bond highlighted in red. Examples of one buyable molecule matching each of the starting materials are displayed in green boxes. (B) Constructed synthetic route with concrete structures, inspired by the higher-level route for pinolidoxin, with the steps corresponding to steps *r*_*i*_ in the higher-level route labeled as *r*_*i*_^′^. The disconnected bonds in these steps are highlighted in red. Note that terminal C^(−)^ in structures **35**, **39**, and **40** was modified to C^(+)^ in the full synthetic route after expert elaboration and refinement.

While the proposed route may already be useful to an experienced chemist, experimental implementation of the proposed routes requires mapping the higher-level molecules back into specific molecules. We selected specific starting materials and incorporated FGIs or protecting groups when necessary, mapping the suggestion by the higher-level algorithm back to full synthetic pathways with concrete molecular structures. Additionally, we allowed for modifications in this stage, such as removing or introducing a strategic step, to refine the proposed routes with synthetic feasibility in mind. This resembles how chemists often adapt retrosynthetic plans during synthesis development, that is, by adjusting or elaborating intermediates when initial disconnections require tactical revision in practice (Section S2).

To illustrate a way that a higher-level route proposed by our algorithm can be mapped to concrete synthesis plans, we construct the route shown in Figure 6B for the forward synthesis of (−)-pinolidoxin, with the higher-level retrosynthesis in mind. By separating strategy from tactical details, our framework enables ideation at a high level, allowing chemists to reintroduce specific functional groups for downstream use while considering feasibility, selectivity, and reactivity. Furthermore, the suggestions generated by our algorithm can inspire creative thinking and the development of novel transformations to realize the overall strategy more effectively. For instance, in this case study, we propose a pyridinium displacement to execute step **xvi** recommended by our algorithm, a transformation that, to our knowledge, has not yet been reported in the literature. Further details on this case study are provided in Section S8.2.1 of the Supporting Information, including a variant of the forward synthesis that employs more conventional transformations (Figure S13).

### Case Study: Pendolmycin

The higher-level algorithm’s capability in identifying multistep routes is further demonstrated in the case study of pendolmycin, an indole alkaloid natural product isolated from *Nocardiopsis strain SA1715* that inhibits epidermal-growth-factor-induced phosphatidylinositol turnover.^61,62^ As in the previous case study, the original algorithm is not able to propose routes for pendolmycin. Informed by the shortest proposed higher-level route in Figure 7A, we present a retrosynthetic analysis of (−)-pendolmycin (**53**) in Figure 7B. The details about the proposed route and additional higher-level routes for pendolmycin can be found in Section S8.2.2 in Supporting Information. Case study results for additional compounds are provided in Section S8.3, and additional results obtained with the higher-level model trained on the Pistachio data set are included in Section S9.

**Figure 7:**
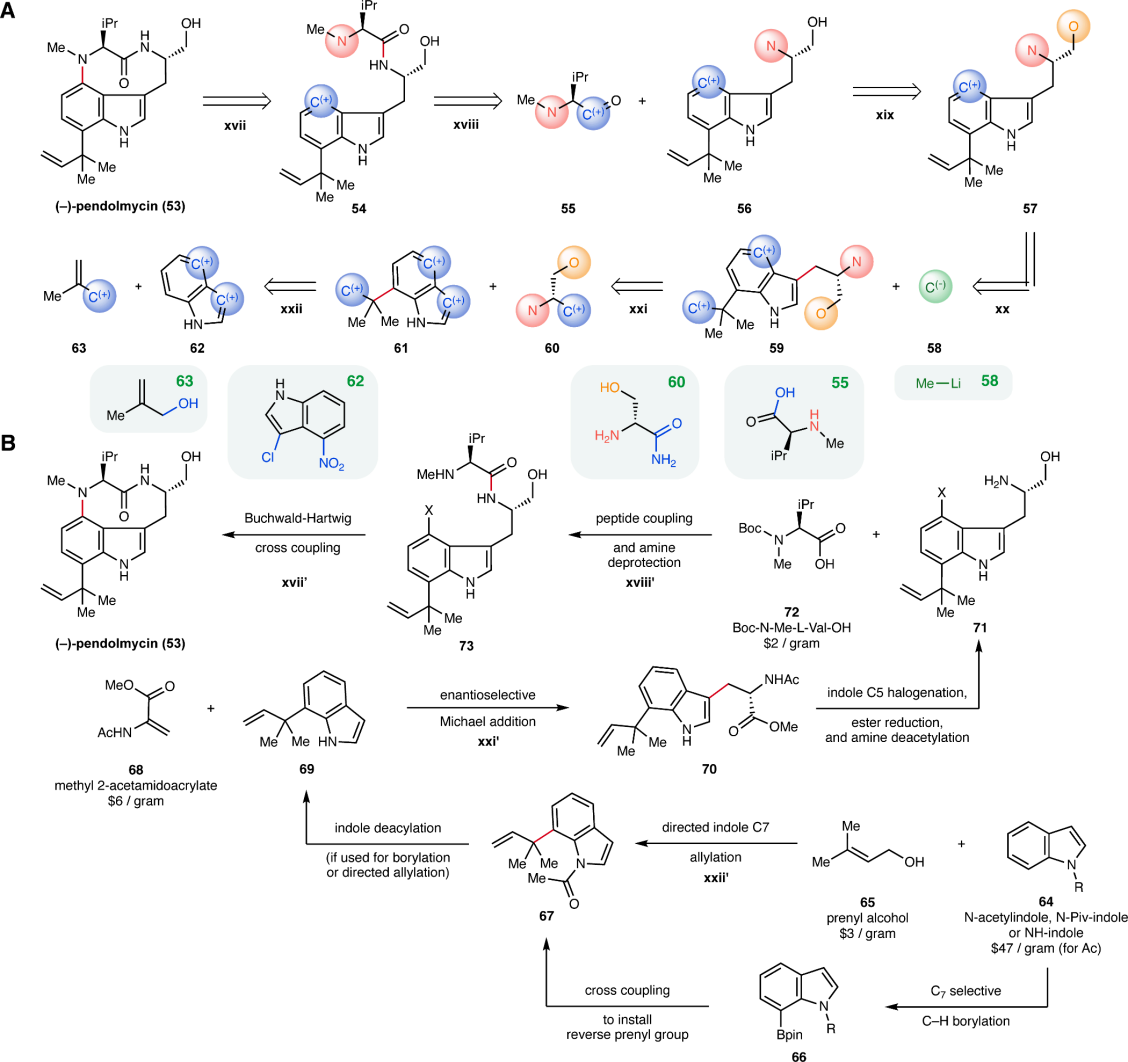
Case study: Pendolmycin. (A) One of the shortest routes proposed by the higher-level algorithm for pendolmycin, with the disconnected bond highlighted in red. Examples of one buyable molecule matching each of the starting materials are displayed in green boxes. (B) Constructed synthetic route with concrete structures, inspired by the higher-level route for pendolmycin, with the steps corresponding to steps *r*_*i*_ in the higher-level route labeled as *r*_*i*_^′^. Note the disconnected bonds in these steps are highlighted in red. When generating this route, disconnection **xx** in the higher-level route has been removed to refine the synthetic strategy.

Importantly, the higher-level retrosynthesis planning algorithm uniquely generates higher-level molecules that encapsulate general reactivity modes without delving into the specifics of strategically equivalent or analogous functional groups. At face value, this feature could be viewed as a limitation, since it therefore requires the chemist to identify which buyables to implement in a forward synthesis. However, to the synthetic chemist, this level of flexibility is a strength of the higher-level algorithm, since abstraction allows synthetic chemists to leverage their expertise and intuition in making informed decisions about functional group identity, organometallic reagents, leaving groups, and other critical components. Unlike traditional algorithms that may impose rigid constraints based on specific substrates or reaction conditions, this approach empowers chemists to consider a broader range of options, tailoring their strategies to the nuances of their target molecules, the complexities of downstream compatibility, and even exploring more creative solutions to carry out the strategy more effectively. Additionally, this approach allows the chemist to select functional groups with the tolerances of the entire synthetic pathway in mind, which typical CASP tools struggle to consider. Ultimately, this flexibility enhances the synthetic design process, allowing for solutions that align with the chemist’s knowledge and the unique challenges presented by each synthesis.

## Conclusion

We present a higher-level retrosynthesis planning algorithm as an approach that aligns with expert chemist strategies and leads to more effective exploration of hypothetical pathways. By abstracting the identities of specific functional groups in the intermediates of a synthesis pathway that do not appear in the final product, our approach encapsulates general reactivity modes without addressing the specifics of strategically analogous functional groups. This formulation allows the algorithm to focus on the broader strategies of synthesis planning revealed by literature pathways and postpone consideration of the tactics for achieving those transformations to a later stage, resulting in improved computational efficiency by reducing the length of the pathways and the branching that is required to capture different reactivities. The empirical success of this approach is demonstrated by the higher success rates in both single-step and multistep planning. Furthermore, through case studies with natural products and drug molecules, we demonstrate how the strategies identified by our framework serve as a powerful foundation for chemists to develop full synthesis plans, which is especially valuable in challenging scenarios where standard CASP methods trained on the same data fail to propose pathways. The flexibility of our approach across diverse functional groups and reactions allows chemists to effectively apply their expertise and intuition in translating these strategies into practical synthetic routes.

There are several opportunities to extend and further develop our higher-level retrosynthesis framework. While demonstrated here with a specific single-step model and multistep search algorithm, alternative model architectures and search strategies (Section S6.2.1) could also be adapted within this framework. In addition, developing an algorithmic process for mapping abstracted intermediates to concrete molecular structures represents an important, complementary direction to be explored in future work. Currently, the higher-level retrosynthesis algorithm operates as a human-machine collaborative tool, where the algorithm proposes higher-level routes (i.e., strategies) and chemists subsequently consider how best to instantiate that pathway. Automating this instantiation step would involve assigning specific functional groups to the higher-level representations and ranking multiple potential structures based on functional group compatibility, reaction feasibility, known reactivity patterns, and possible functional group interconversions, potentially guided by reaction outcome prediction models. Additionally, as retrosynthesis in practice may be more dynamic and iterative than our current approach allows, this framework could be extended to incorporate a refinement module that revises or rearranges strategies proposed by our algorithm based on downstream feasibility, dynamically alternating between addressing considerations of strategy and considerations of tactics in an iterative manner. Doing so will enable the generation of full synthetic routes while maintaining the benefits of this higher-level approach, ultimately providing even stronger decision-making support to chemists in pursuit of novel, complex molecules.

## Supplementary Material



## Notes

Data and code availability, examples of retrosynthetic strategies and tactics used by chemists, complete computational methods and implementation details for reaction cleaning and abstraction, data set construction, model training, details on the multistep planning algorithm, benchmarking and evaluation procedures, additional computational results and case studies, generalization to larger data sets, and a discussion on the role of experimental validation in the context of this study (PDF)

## ‡

(J.F.J.) Kookmin University, Seoul, 02707, South Korea. Work done while at the Massachusetts Institute of Technology

## ⊥

(G.L.B.) Merck, South San Francisco, California 94080, United States. Work done while at the University of California, Berkeley.

## m

Department of Electrical Engineering and Computer Science, Massachusetts Institute of Technology, Cambridge, Massachusetts 02139, United States.

## h

Department of Computer Science, Duke University, Durham, North Carolina 27705.
