# Supplementary material for: Higher-Level Strategies for Computer-Aided Retrosynthesis
Source: ACS Cent Sci. 2026 Mar 5;12(3):345–57. doi: 10.1021/acscentsci.5c02014 (PMC13022727; doi:10.1021/acscentsci.5c02014)
Supplement: Supplementary file 1 [file oc5c02014_si_001.pdf]

Supporting Information: Higher-Level Strategies for Computer-Aided Retrosynthesis

Jihye Roh,[†] Joonyoung F. Joung,[†,‡] Kevin Yu,[¶,§] Zhengkai Tu,[‖] G. Logan Bartholomew,[⊥,#] Omar A. Santiago-Reyes,[@] Mun Hong Fong,[†,△] Richmond Sarpong,[⊥] Sarah E. Reisman,[@] and Connor W. Coley[*,†,‖]

†*Department of Chemical Engineering, MIT, Cambridge, MA 02139, USA*

‡*Current affiliation: Kookmin University, Seoul, 02707, South Korea. Work done while at MIT*

¶*Center for Computational Science and Engineering, MIT, Cambridge, MA 02139, USA*

§*Current affiliation: Department of Electrical Engineering and Computer Science, MIT, Cambridge, MA 02139, USA*

‖*Department of Electrical Engineering and Computer Science, MIT, Cambridge, MA 02139, USA*

⊥*Department of Chemistry, University of California, Berkeley, Berkeley, CA 94720, USA*

#*Current affiliation: Merck, South San Francisco, CA 94080, USA. Work done while at the University of California, Berkeley*

@*Division of Chemistry and Chemical Engineering, California Institute of Technology, Pasadena, CA 91125, USA*

△*Current affiliation: Department of Computer Science, Duke University, Durham, NC 27705. Work done while at MIT*

E-mail: ccoley@mit.edu

Contents

S1 Data and model availability **S4**

S2 General retrosynthetic strategy and tactics **S5**

S3 Reaction classification, cleaning, and processing **S6**

S4 Curating the higher-level reaction and route datasets **S7**

S4.1 General abstraction heuristics and representations S7

S4.2 Exceptions and additional details about abstraction heuristics S9

S4.3 Generating the higher-level route datasets S10

S4.4 Example original and higher-level routes S11

S4.5 Statistics and examples of abstracted groups S16

S5 Developing the higher-level retrosynthetic planning algorithm **S20**

S5.1 Higher-level single-step retrosynthesis model S20

S5.1.1 Extracting and consolidating templates S20

S5.1.2 Template consolidation results S22

S5.1.3 Training the higher-level single-step model S23

S5.1.4 Details on single-step retrosynthesis model performance S25

S5.2 Developing the higher-level multistep retrosynthesis algorithm S27

S5.2.1 Details on selection, expansion, and update steps in MCTS S27

S5.2.2 Details on higher-level multistep retrosynthesis algorithm S29

S6 Evaluating the higher-level retrosynthesis algorithm **S31**

S6.1 Evaluation setup and parameters S31

S6.2 Benchmarking on USPTO-190 test molecules S32

S6.2.1 Contextualization relative to multistep retrosynthesis algorithms with value functions S35

S7 Evaluating feasibility of matched buyable molecules **S36**

S8 Case studies with drugs and natural products **S40**

S8.1 Overview of case-study results S41

S8.2 Additional details on main-text case studies S43

S8.2.1 Case study: Pinolidoxin S43

S8.2.2 Case study: Pendolmycin S47

S8.3 Additional Case Studies S49

S8.3.1 Additional case study: Illudinine S50

S8.3.2 Additional case study: Albocycline S51

S8.3.3 Additional case study: Madumycin I S52

S9 Generalizing to the larger, more diverse Pistachio dataset **S53**

S9.1 Template extraction, single-step model training and performance S54

S9.2 Case studies with Pistachio models S56

S9.2.1 New routes to previously solvable targets S57

S9.2.2 Additional solvable targets S58

S10 Comments on the role of experimental validation **S60**

References **S62**

S1 Data and model availability

Source code for curating the dataset, training single-step models, and performing retrosynthetic planning can be found in the following GitHub repository: https://github.com/jihye-roh/higherlev_retro. This repository includes a detailed README with instructions on how to reproduce the results in this manuscript. Datasets curated from the USPTO-Full[1] dataset and models trained on this data are available at https://doi.org/10.6084/m9.figshare.28306673. The Pistachio dataset[2] is commercially-licensed from NextMove Software and cannot be released.

S2 General retrosynthetic strategy and tactics

Fig. S1 illustrates how retrosynthetic planning often prioritizes strategic disconnections before fully resolving tactical details such as functional group compatibility. When translating a retrosynthetic plan into a forward synthesis, chemists must consider various tactics to implement the overarching strategy. A single retrosynthetic transformation can correspond to multiple steps in the forward direction, and additional modifications are often necessary to enable key transformations.

In the retrosynthetic analysis for cotylenol, the aglycone of (+)-cotylenin A, the authors initially proposed using a SO_2Mes leaving group for the key coupling between the A and C ring fragments. However, this approach proved unsuccessful under a range of reaction conditions, including the use of reducing agents. As a result, the authors pivoted to using an α-bromo ketone in an Utimoto coupling, which successfully afforded the desired product.[3]

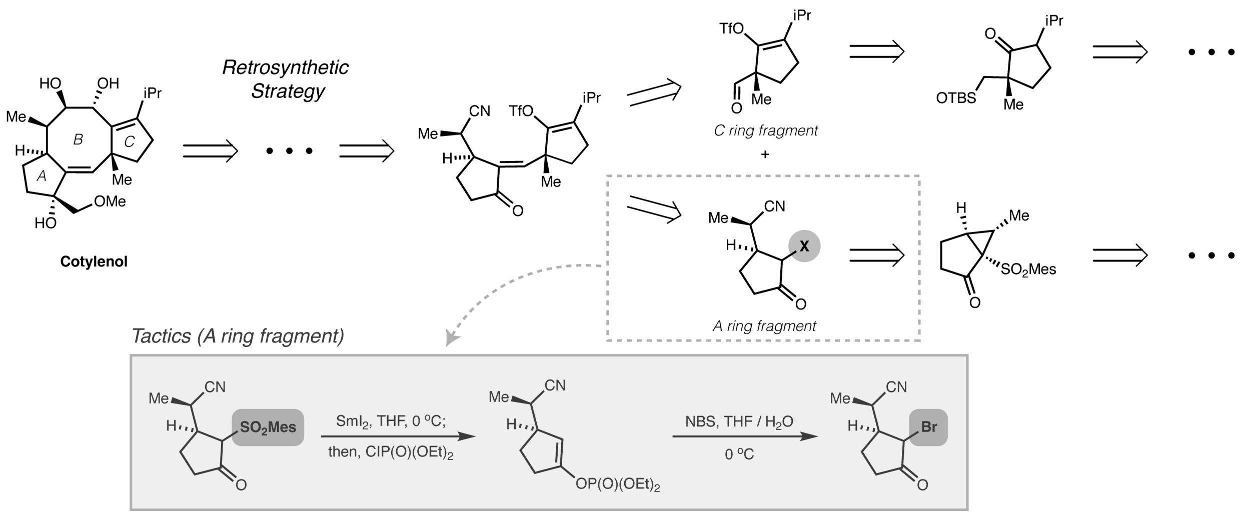

Figure S1: Selected steps in the retrosynthesis and forward synthesis of cotylenol by Uwamori et al.[3]. The initial retrosynthesis required tactical adjustments when implementing in the forward synthesis.

S3 Reaction classification, cleaning, and processing

We use the simplified molecular input line entry system (SMILES),[4] a machine-readable line-notation for chemical structures, to represent molecules and reactions. The SMILES string of reactants, reagents, and products are concatenated with `>` between each field to form the SMILES string of reactions (e.g., `REACTANTS>REAGENTS>PRODUCTS`), where different species in each field are separated with a dot. The SMILES string of reactions can also contain isotope labels located in front of atomic species or numbers mapping the reactant atoms to product atoms (i.e., atom-mapping).

We used the USPTO-Full dataset,[1] parsed as described in Coley *et al.*[5] The resulting reactions were classified and re-atom mapped using NextMove's NameRxn software[6] for consistent atom-mapping. Unclassified reactions were removed from the dataset as NameRxn does not generate any atom mapping, which is essential in the higher-level dataset generation process, for these reactions. Then, multi-product reactions were separated into multiple single-product reaction entries with the same reactants and reagents. Each single-product reaction was further processed by filtering and recategorizing chemical species (reactants, products, and reagents) based on atom mapping. Reagents containing atom mappings were first moved to the reactant side. Then, products were removed if they had more than 5 heavy atoms (i.e., non-H atom) without a corresponding atom on the reactant side. Common species between the reactants and products, identified after neutralizing and stripping stereo-chemistry information, were recategorized as reagents. We also ensured that each product shares at least one atom mapping with the reactants, and vice versa; any reactant species not meeting this criterion was recategorized as a reagent, while products were removed if they did not satisfy this requirement. Reactions without reactants or products after this recategorization were filtered out. Finally, the reactions in the USPTO-190 dataset[7] were filtered out, since the target molecules in these routes are used to evaluate the multistep planning performance.

S4 Curating the higher-level reaction and route datasets

S4.1 General abstraction heuristics and representations

Inspired by synthons, our higher-level representation of molecules involve abstracting the substructures of the molecule that do not contribute to the structure of the reference molecule (e.g., final target product). The atom mapping in the reference molecule is used to identify core atoms/substructures (i.e., each atom is present in the reference molecule) and leaving groups in the current molecule. The leaving group and its connected core atom are then abstracted into a single atom-style group representation, with the core atom's identity preserved and the structure represented by isotope labels on that atom.

For a molecule $R_1 - X - Y - LG$, where the core scaffold $R_1 - X$ is connected to the leaving group $Y - LG$ through a bond between atoms X and Y, the abstracted representation becomes $R_1{-}^{a}\mathrm{X}$, where a denotes the isotope label used to represent abstracted groups. Note that bonds are not necessarily single bonds and implicit hydrogen is not specified in this notation. The group label (i.e., isotope a) is determined by the identity of X and the electronegativity of Y. The final target molecule in the multistep route is generally used as a reference when identifying the leaving atoms/substructures. The value of a depends on the identity of X and the electronegativity of Y. In the SMILES string, this is expressed as `[aXHn]`, where n is the number of implicit hydrogen atoms.

When X is a heteroatom, the groups are differentiated only by the identity of X. That is, the molecule is abstracted as $R_1{-}^{1}\mathrm{X}$, regardless of Y (including hydrogen). When X is a carbon atom, the abstracted groups are divided into multiple subgroups depending on the electronegativity of leaving groups to better capture the reactivity. Each subgroup is represented with a different isotope label. If Y is also a carbon atom, then the groups are further divided into three subgroups depending on the bond between X and Y, where single-, double-, and triple- bonds are each represented as $R_1{-}^{1}\mathrm{C}$, $R_1{-}^{2}\mathrm{C}$, and $R_1{-}^{3}\mathrm{C}$, respectively. If Y is more electronegative than C, then the molecule is abstracted as $R_1{-}^{4}\mathrm{C}$

(i.e., $R_1{-}C^{(+)}$). If Y is less electronegative than C (excluding hydrogen), then the molecule is abstracted as $R_1{-}{}^5C$ (i.e., $R_1{-}C^{(-)}$). In the SMILES string, isotope labels are used for consistency. If Y is a hydrogen atom, we choose not to abstract it and keep its identity. When X is bonded to multiple leaving atoms, the group with higher isotope-label value is given priority.

Isotope labels are excluded from the figures for clarity, with abstracted groups represented as spheres or ellipsoids around the core atom. ${}^1C$, ${}^2C$, and ${}^3C$ are illustrated with ellipsoids around the corresponding carbon atoms, showing the specific bond types. ${}^4C$ and ${}^5C$ are depicted as $C^{(+)}$ and $C^{(-)}$ in spheres, respectively.

S4.2 Exceptions and additional details about abstraction heuristics

We choose not to abstract the leaving atoms/substructures in certain cases. A single leaving heavy-atom is not abstracted if it contributes to the aromaticity of the molecule (e.g., 4-pyrone to 4-pyridone reaction). If abstracting the molecule leads to the molecule breaking into multiple fragments of core scaffolds, we do not abstract the atom. Methyl ketone demethylation or 1,2-diol to aldehyde reactions are not abstracted. During higher-level route generation, intermediates with unabstracted leaving heavy atoms were designated as new references for their successors, with their abstracted groups propagated, to ensure atom continuity throughout the pathway.

(Thio)-ketal or acetal protected carbonyls are abstracted as $R_1{-}^1\mathrm{C}{=}^1\mathrm{O}$, using both C and O atoms to represent the abstracted structure. If the leaving substructures are $-\mathrm{C}\equiv\mathrm{N}$ or $-\mathrm{CX}_3'$ (X′ is F, Cl, Br, or I) with a single bond between carbon X and carbon Y, we classify this as an electrophilic carbon group $R_1{-}\mathrm{C}^{(+)}$. When Y is a carbonyl C, we abstract this group as a nucleophilic carbon group $R_1{-}\mathrm{C}^{(-)}$.

Aliphatic atoms representing abstracted groups are neutralized to enhance the generality of the representations, except when bonded to a non-leaving atom with an opposing charge. For example, nitro groups that are subsequently reduced to form the target product (i.e., with the departing oxygen atoms) are abstracted as neutral N groups, similar to the abstraction of protected amine groups.

S4.3 Generating the higher-level route datasets

The processed reactions were grouped by patent, and multistep routes were extracted from each patent following the workflow of Mo *et al.*[8] Each extracted route was represented as a directed tree, with molecules as nodes and edges directed from the product to the corresponding reactants. Atom mapping was propagated from the target molecule down the synthesis tree using atom mapping in the reaction SMILES to trace each atom along the pathway.

With the extracted routes, we generated corresponding higher-level routes by abstracting individual chemical nodes using the abstraction heuristics, generally using the target molecule as a reference except in specific cases noted in Section S4.2. When multiple nodes in the synthesis pathway were abstracted to the same representation, any reaction between the nodes were removed, making the synthesis pathways shorter. Any chemical node that does not contribute at least one atom to the reference molecule were also removed from the synthesis tree. Reaction SMILES were collected from the resulting trees by combining the SMILES of a molecule with the SMILES of its children nodes in the form `{SMILES_OF_CHILD_1}{SMILES_OF_CHILD_2} ... »{SMILES_OF_CURRENT_NODE}` to construct the higher-level reaction dataset.

Examples of the resulting abstracted routes are included in Section S4.4, and statistics on the abstracted functional groups are summarized in Section S4.5.

S4.4 Example original and higher-level routes

Figs. S2-S5 show examples of multistep routes extracted from the original reaction dataset and the corresponding higher-level routes generated by applying the abstraction heuristics. The target molecule is used as a reference when generating all abstractions unless otherwise mentioned. Note that *tactic*-al reactions, such as deprotection steps, are eliminated in this abstraction process only when they change leaving atoms/substructures, that is, groups that have been abstracted previously.

*Multistep route extracted from the original reaction dataset (**Original** route)*

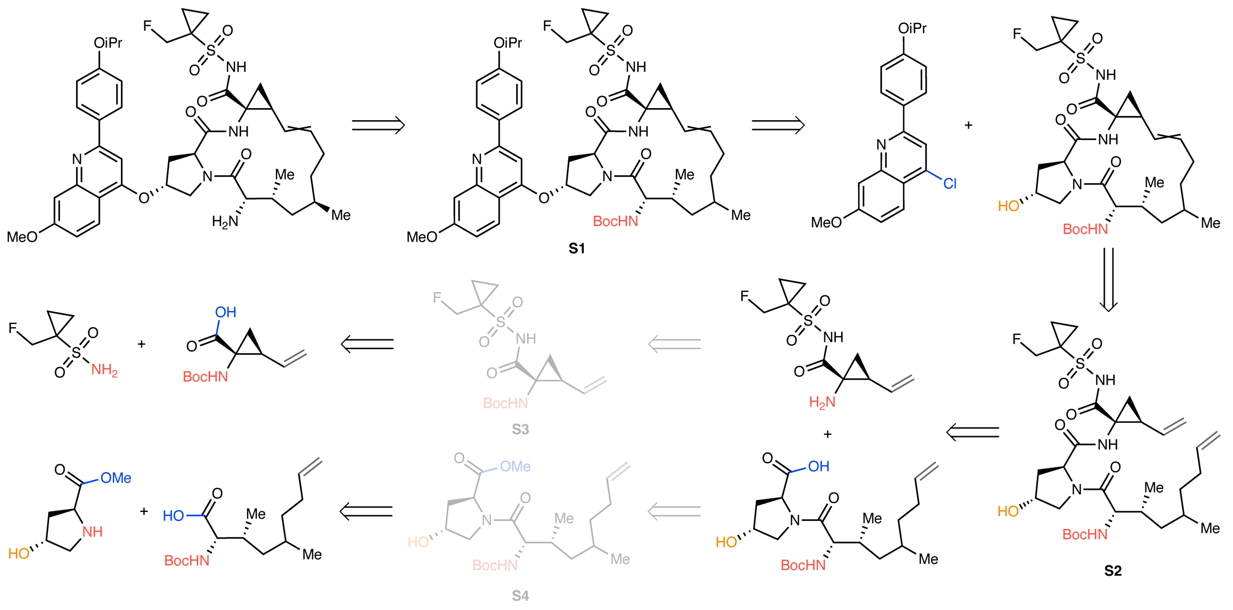

***Higher-level route** generated using the abstraction heuristics*

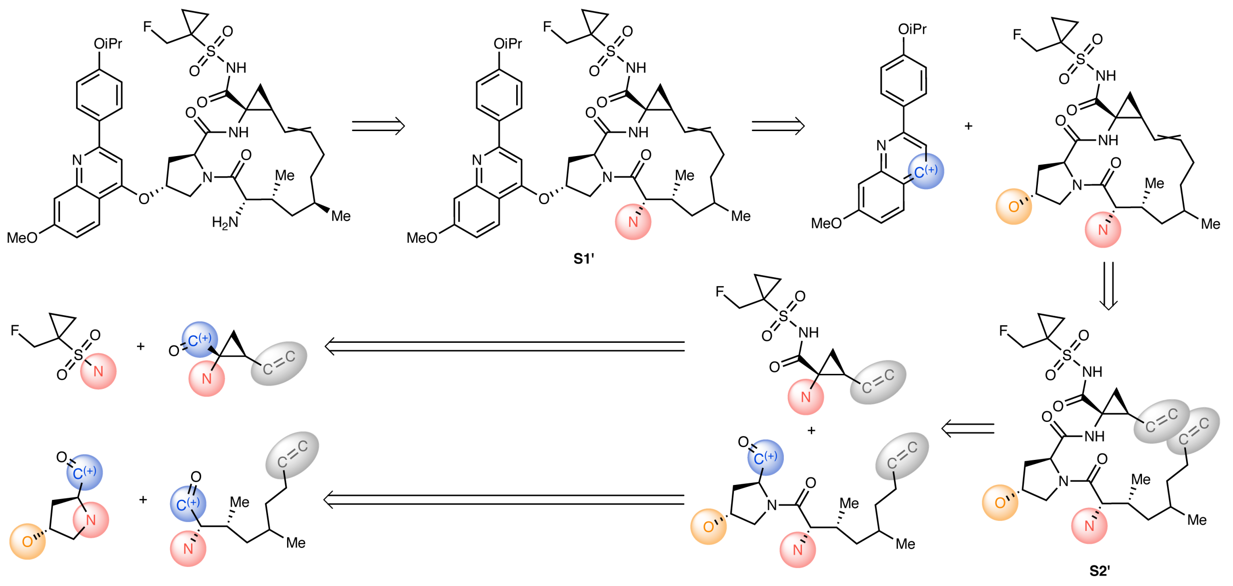

Figure S2: General route abstraction example. The original route (top) and the corresponding higher-level route (bottom) generated with the abstraction heuristics are shown. Molecules in the higher-level route align with their counterparts in the original route, with removed molecules and reactions shown in gray. The routes are truncated to highlight key intermediates, such as **S2** in the olefin metathesis and its higher-level equivalent **S2'**. Note the abstraction of **S1** adds **S1'** as an intermediate in the higher-level route as the N atom in the amine group exists in the final product. This is different from how abstracting **S3**, with a similar Boc-protected amine group, or **S4** do not add new intermediates, removing the deprotection steps in the higher-level route.

*Multistep route extracted from the original reaction dataset (**Original** route)*

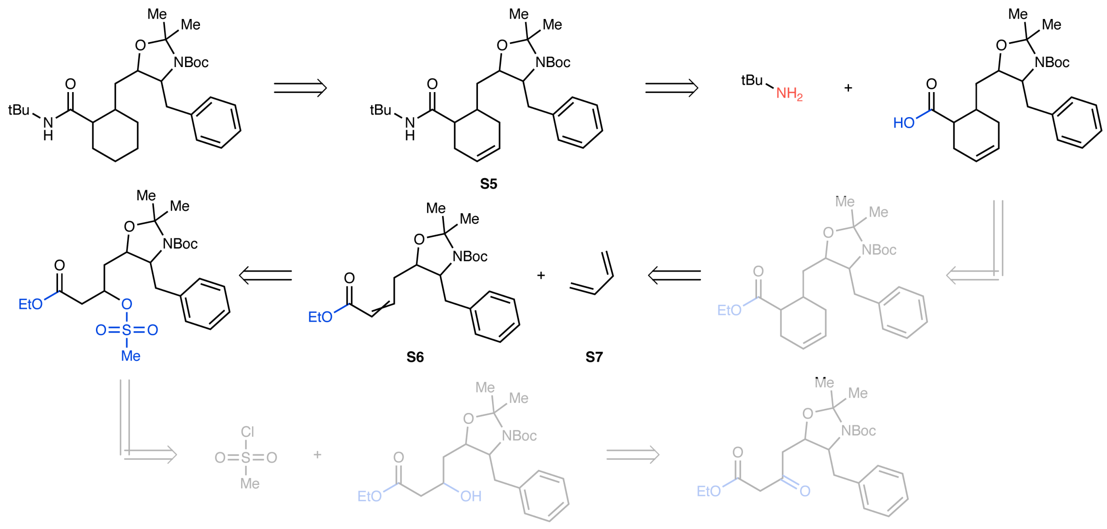

***Higher-level** route generated using the abstraction heuristics*

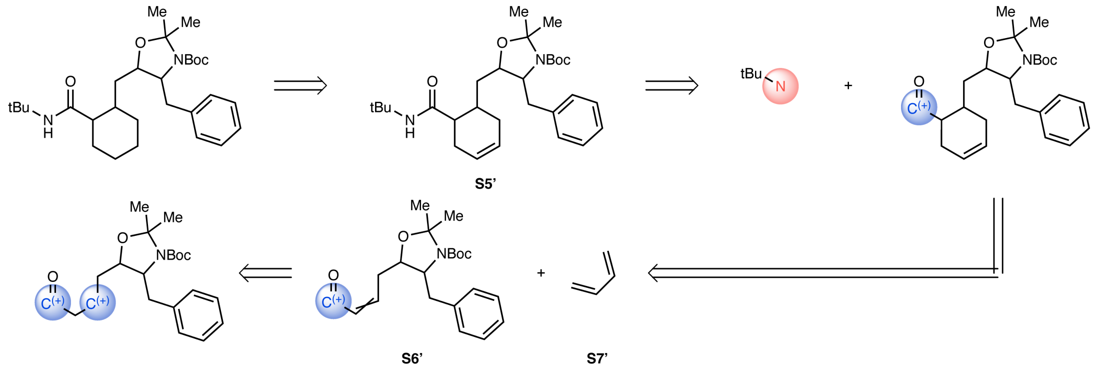

Figure S3: Route abstraction example: Intermediates without leaving atoms. The original route (top) and the corresponding higher-level route (bottom) generated with the abstraction heuristics are shown. The alkene **S5**, which is halogenated to form the target product, and **S7**, which undergo the Diels-Alder cycloaddition with **S6**, do not have leaving atoms. As a result, they are not abstracted in the higher-level route (**S5'** and **S6'** are equivalent to **S5** and **S6**, respectively).

*Multistep route extracted from the original reaction dataset (**Original** route)*

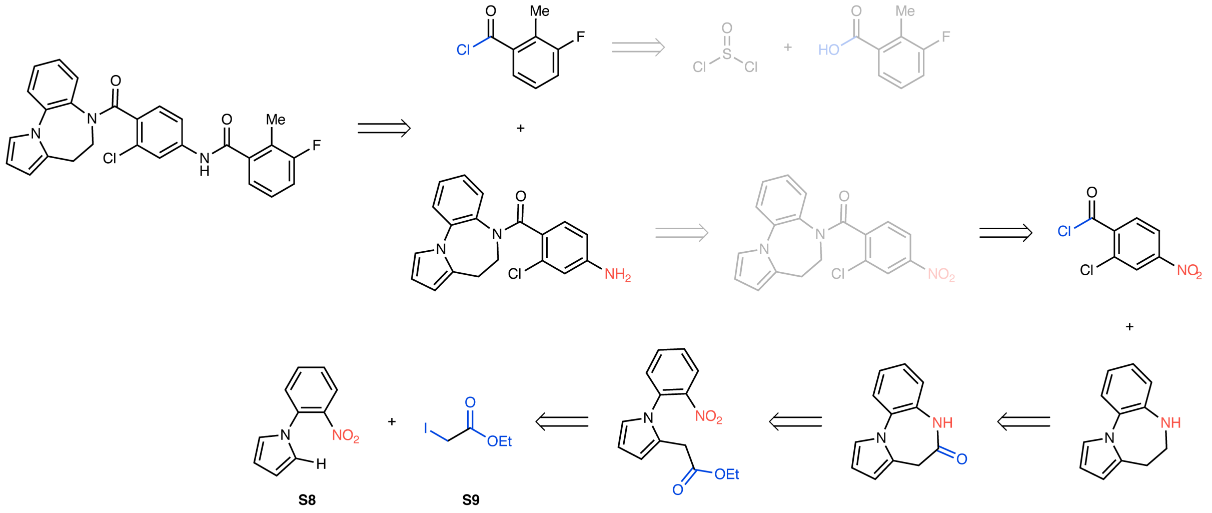

***Higher-level** route generated using the abstraction heuristics*

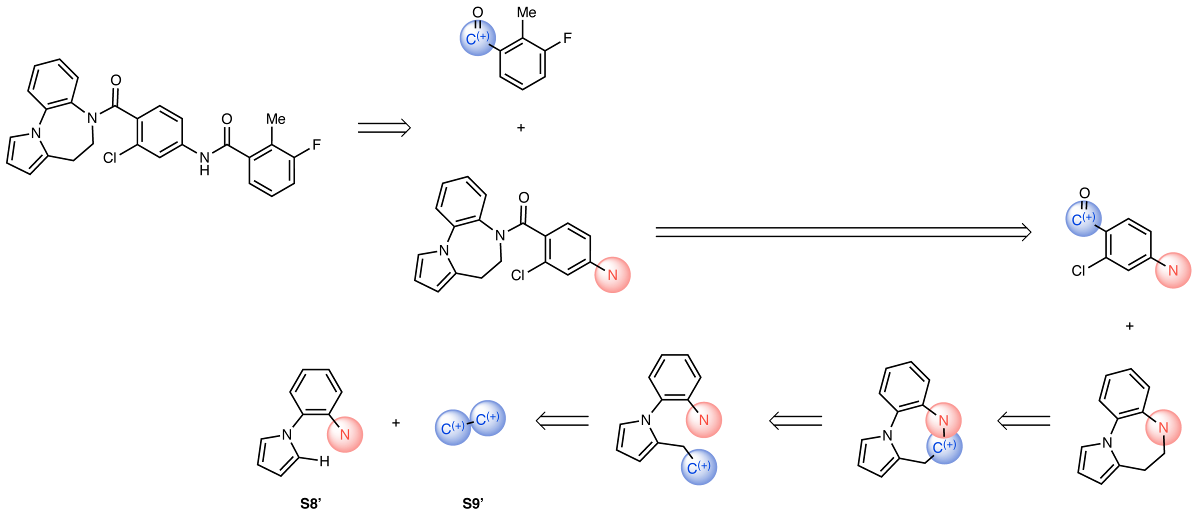

Figure S4: Route abstraction example: C-H functionalization. The original route (top) and the corresponding higher-level route (bottom) generated with the abstraction heuristics are shown. The leaving hydrogen atom in **S8**, which undergo Minisci reaction with **S9**, is retained without being abstracted, as shown in **S8'**.

*Multistep route extracted from the original reaction dataset (**Original** route)*

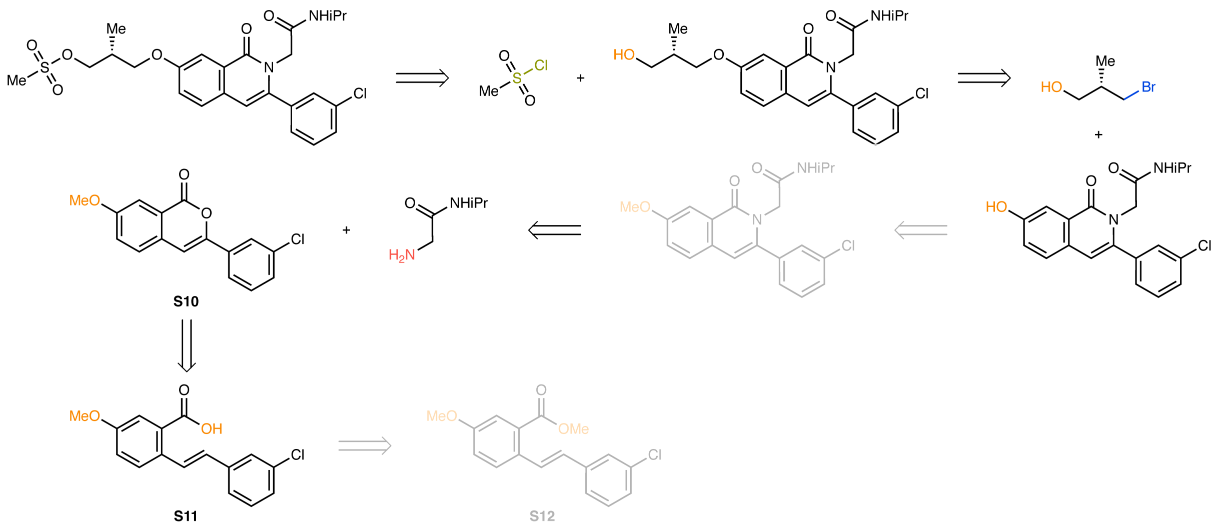

***Higher-level** route generated using the abstraction heuristics*

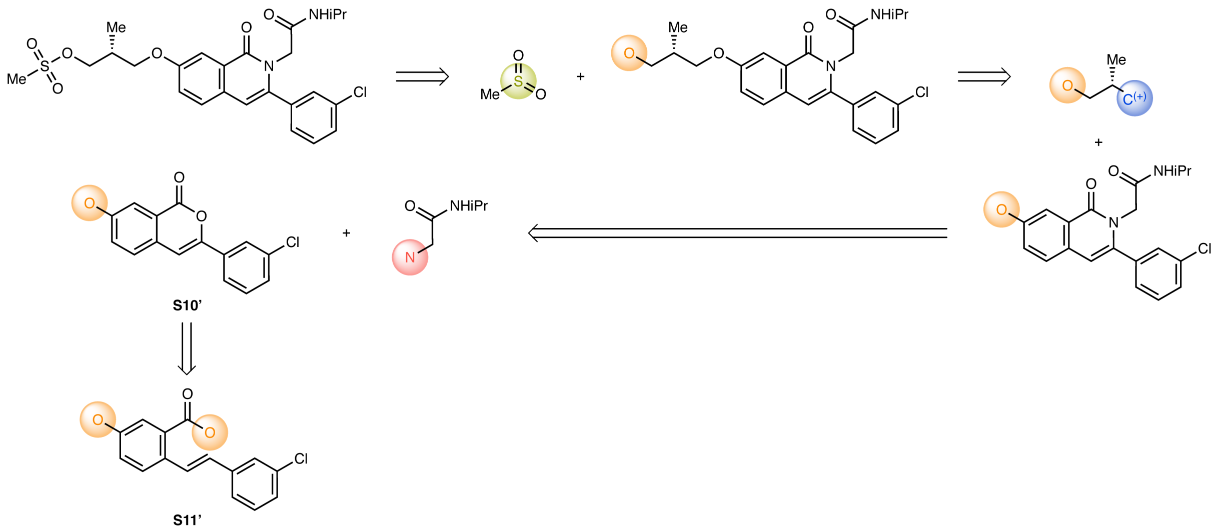

Figure S5: Route abstraction example: exception due to aromaticity. The original route (top) and the corresponding higher-level route (bottom) generated with the abstraction heuristics are shown. The leaving aromatic oxygen atom in intermediate **S10** is not abstracted in **S10'**. Therefore, the target molecule is used as a reference when generating abstractions until **S10**, and **S10'** is used as a new reference when generating abstractions for **S11** and **S12**.

S4.5 Statistics and examples of abstracted groups

The statistics on abstracted groups and representative examples for heteroatom- and carbon-based groups are summarized in Tables S1 and S2, respectively. Fig. S6 illustrates selected examples of abstracted groups and their corresponding functional groups from Tables S1 and S2. Statistics were computed prior to removing any reactions between nodes abstracted to the same representation, in order to fully capture the diversity of functional groups. A total of 21 distinct abstracted groups were identified, without distinguishing between aromatic and aliphatic forms.

Table S1: Statistics of abstracted heteroatom groups and examples of common functional groups/species. Occurrences are counted across all molecules in all routes, with multiple instances within the same molecule counted individually. Abstracted groups are each sorted in descending order of occurrence. For each abstracted group, five representative examples of original functional groups or species that map to it are shown. Note that the examples also include the core atom connected to the leaving group. Functional groups are typically connected to other core structures, while species are single-atom groups in which all of the other structure leaves. Dashes (−) are placed in front of functional groups to distinguish them from full species and are not meant to indicate bond order or connectivity. [H] or [H_n] ($n = 1, 2$) denote optional hydrogen atoms that may be present depending on how the abstracted group is connected to the remaining structure. For instance, −N[H]Boc includes both R_2NBoc and RNHBoc, where R is the remaining structure in the higher-level molecule. The common functional groups may originate from common reagents used to install specific functional groups. For instance, −BBpin is from when B_2pin_2 is used to install a −Bpin group and $-SnSnMe_3$ is from when $(SnMe_3)_2$ is used to install a $-SnMe_3$ group. Cases where only hydrogen atoms are abstracted (e.g., $-NH_x$ for ^{1}N) are excluded. See Fig. S6 for examples.

Group	Occ.	Examples of common functional groups/species				
^{1}N	991k	$-N_3$	$-NO_2$	−N[H]Boc	−N[H]Bn	−N[H]Cbz
^{1}O	447k	−OMe	−OEt	−O*t*Bu	−OBn	$-OSiMe_2t$Bu
^{1}S	68.4k	−SCl	−SAc	−SOTf	P_4S_{10}	Lawesson's reagent
^{1}Cl	16.0k	$SOCl_2$	SO_2Cl_2	$POCl_3$	$(COCl)_2$	NCS[1]
^{1}Br	15.5k	Br^-	Br_2	PBr_3	CBr_4	NBS[1]
^{1}Si	5.75k	−SiF	−SiCl	−SiBr	−SiOTf	$-SiNSiMe_3$
^{1}I	4.12k	I^-	I_2	CH_2I_2	$ISiMe_3$	NIS[1]
^{1}B	2.97k	−BBpin	−BOMe	−BOEt	−BOiPr	−BO*n*Bu
^{1}F	2.18k	F^-	F_2	DAST[2]	NFSI[3]	Species 1[4]
^{1}P	1.20k	$-PO^-$	−POH	−PCl	−POEt	−PO*n*Bu
^{1}Sn	657	−SnCl	−SnBr	−SnI	$-SnSnMe_3$	$-SnSnBu_3$
^{1}Li	88	*n*-BuLi	*t*-BuLi	·	·	·
^{1}Se	42	−SeCl	−SeBr	−SeCN	$-SeO_2$	−SePh
^{1}As	2	AsI	·	·	·	·
^{1}Sb	1	$SbCl_2$	·	·	·	·

[1] NCS: *N*-chlorosuccinimide, NBS: *N*-bromosuccinimide, NIS: *N*-iodosuccinimide
[2] DAST: Diethylaminosulfur trifluoride
[3] NFSI: *N*-Fluorobenzenesulfonimide
[4] Species 1: 1-chloromethyl-4-fluoro-1,4-diazoniabicyclo[2.2.2]octane

Table S2: Statistics of abstracted carbon groups and examples of common functional groups. Occurrences are counted across all molecules in all routes, with multiple instances within the same molecule counted individually. Carbon-only abstracted groups are each sorted in descending order of occurrence. For each abstracted group, three representative examples of original functional groups are shown. Dashes (−) are placed in front of functional groups to distinguish them from full species and are not meant to indicate bond order or connectivity. [H] or [H_n] ($n = 1, 2$) denote optional hydrogen atoms that may be present depending on how the abstracted group is connected to the remaining structure. S denotes the stereochemistry of the chiral carbon in the functional group.

Group	Occ.	Examples of common functional groups		
^{4}C ($C^{(+)}$)	1.35M	−C[H_n]OH	−C[H]=O	−C[H_n]Br
^{5}C ($C^{(-)}$)	125k	−C[H_n]Cu	−C[H_n]MgBr	−C[H_n]$B(OH)_2$
^{2}C (C=C)	5.50k	−C[H]=CH_2	−C[H]=CHMe	−C[H]=$CHCH_2Br$
^{1}C (C−C)	403	−C[H_n]−CH=CH_2	−C[H_n]−$CH(Me)_2OH$	−C[H_n]−C(OH)Ph (S)
^{3}C (C≡C)	60	−C≡CH	−C≡CMe	−C≡$CSiMe_3$
^{1}C=^{1}O	6.68k	−$C(OMe)_2$	−$C(OMe)_2$	1,3-dioxolane

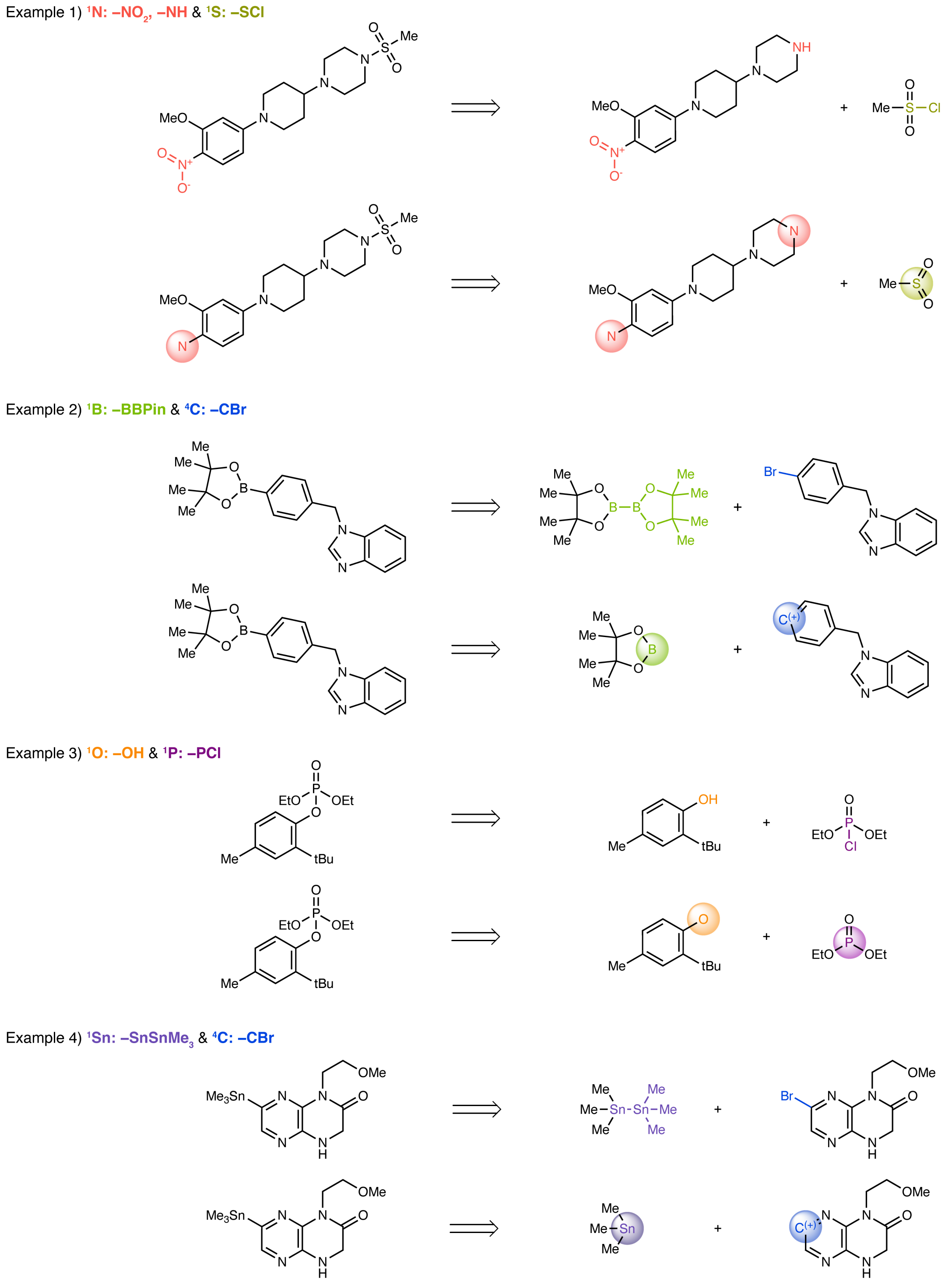

Figure S6: Examples of abstracted groups and corresponding functional groups. Examples of original reactions and higher-level reactions are shown. These examples illustrate additional abstracted groups and demonstrate how the corresponding functional groups are recorded in Tables S1 and S2.

S5 Developing the higher-level retrosynthetic planning algorithm

S5.1 Higher-level single-step retrosynthesis model

S5.1.1 Extracting and consolidating templates

We chose to use a template-based approach for the single-step model, training a feedforward neural network to predict templates given the molecular fingerprint of the product. Templates were extracted from the reactions in the higher-level and original datasets using a modified template extractor in RDChiral[5] that include isotope labels in the atom patterns. Any atom with a change in isotope labels (i.e., abstracted groups) was considered as a changed atom to be included in the template, including the conversion of an abstracted group to a non-abstracted atom or vice-versa, in which the an isotope label of 0 (i.e., no label) was specified in the template for the non-abstracted atom.

Varying levels of template specificity can unnecessarily increase the total number of templates. Additionally, symmetry in reaction center structures may result in duplicate reaction templates with different orders of atom-mapping. Having multiple duplicate and/or nonexclusive templates for the same chemistry makes it difficult for the model to learn from the data, decreasing the accuracy of model predictions and applicability of highly-ranked templates.[9] To address this, we consolidated the templates that encode same the same chemistry into a single, more general template. All extracted templates were collected and applied to the product of each reaction, keeping a record of all templates that successfully recovered the recorded reactant(s). The most frequently appearing (i.e., general) template was kept as the label for each reaction to use in training the single-step model (Fig. S7). No minimum frequency threshold was enforced for the templates; all templates that successfully recovered the recorded reactants were retained. Reactions without a template that recovers the recorded reactants were filtered from the dataset.

To evaluate the affect of template consolidation on performance, we trained two single-step retrosynthesis models for each dataset; one with template consolidation (denoted as *with consol.*) and one without (denoted as *wo consol.*). The same train/validation/test split was used for both the consolidated and non-consolidated models within the same dataset.

S5.1.2 Template consolidation results

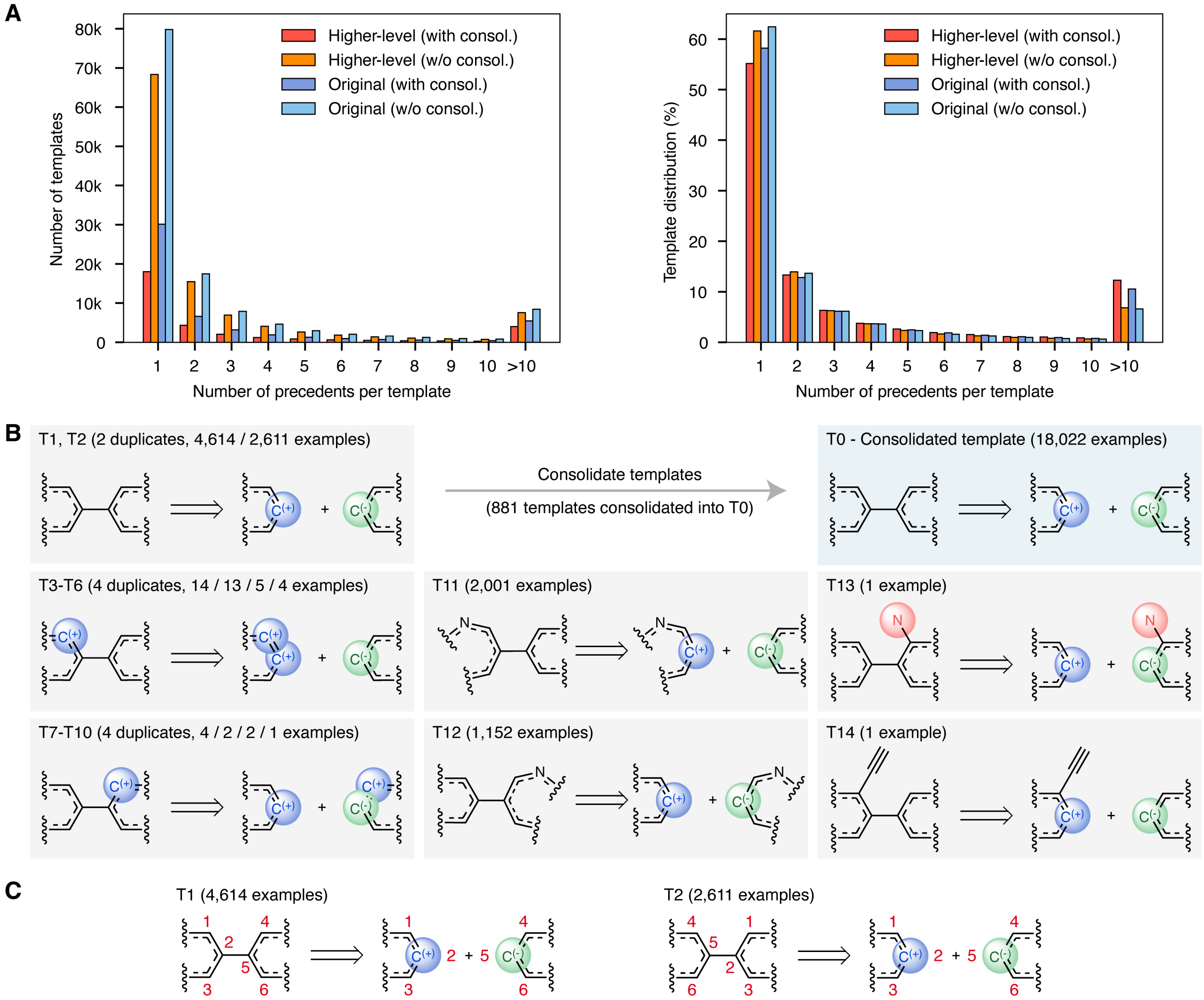

Figure S7: Template consolidation results. (A) The distribution of the number of literature precedents (i.e., reactions) per template. The distribution shifts higher with template consolidation for both the higher-level and original datasets, with a significant decrease in total number of templates (left). The percentage of 1-2 example templates also decrease, indicating that the reactions encoded by some of the rare templates can be covered by the more general templates (right). (B) Examples of templates extracted by RDChiral in the higher-level dataset (T1-T14) that were consolidated into the same template (T0). Duplicate templates and/or more specific templates, which included more abstracted groups or atoms, were consolidated into one, more general template encoding the same chemistry. Note that single-example templates (T13, T14) were also consolidated into T0. (C) Symmetry in the structure lead to duplicate templates with different atom mapping (numbers in red) from RDChiral, which were consolidated to one template.

S5.1.3 Training the higher-level single-step model

All single-step retrosynthesis models are multilayer perceptron (MLP) classification models predicting the best template(s) for each product input. The input for the model is a 2048-bit Morgan Fingerprint representation of the product molecule with radius 2, as implemented in RDKit.[10] The output is a vector with length n_t corresponding to the number of templates. Softmax is applied to the final layer, returning scores (i.e., probabilities) for each template that sum to 1. The highest ranking templates are applied to the product using RDChiral to generate precursors.

All models were trained using the Adam optimizer with early stopping (min delta 10^{-4}, patience 2) with a maximum of 150 epochs, cross-entropy loss, decayed learning rate on plateau (patience 1, factor 0.3), and train/validation batch sizes of 2048. The hyperparameters for each model were determined through a Bayesian search of the following parameter space using the wandb library,[11] selected based on highest accuracy on the validation set (Table S3):

1. Dropout: [0.1, 0.2, 0.3, 0.4, 0.5]
2. Learning rate: [0.00005 - 0.005]
3. Hidden activation: [ReLU, ELU]
4. Number of hidden layers: [1, 2, 3]
5. Hidden units: [512, 1024, 2048]

Hyperparameter optimization and model training were performed on a single NVIDIA GeForce RTX 4090 GPU. The hyperparameters selected for each single-step model are summarized in Table S3 below.

Table S3: Hyperparameters used to train each single-step model.

	Higher-level		Original	
	with consol.	w/o consol.	with consol.	w/o consol.
Dropout	0.5	0.5	0.1	0.1
Learning Rate ($\times 10^{-3}$)	2.52468	3.66103	1.34474	0.44015
Hidden Activation	ReLU	ELU	ReLU	ReLU
Number of Hidden Layers	1	1	1	1
Hidden Sizes	2,048	512	1,024	2,048
Input Dimension	2,048	2,048	2,048	2,048
Output Dimension[1]	32,622	110,929	51,736	127,852

[1]Output dimension is equal to the number of templates.

S5.1.4 Details on single-step retrosynthesis model performance

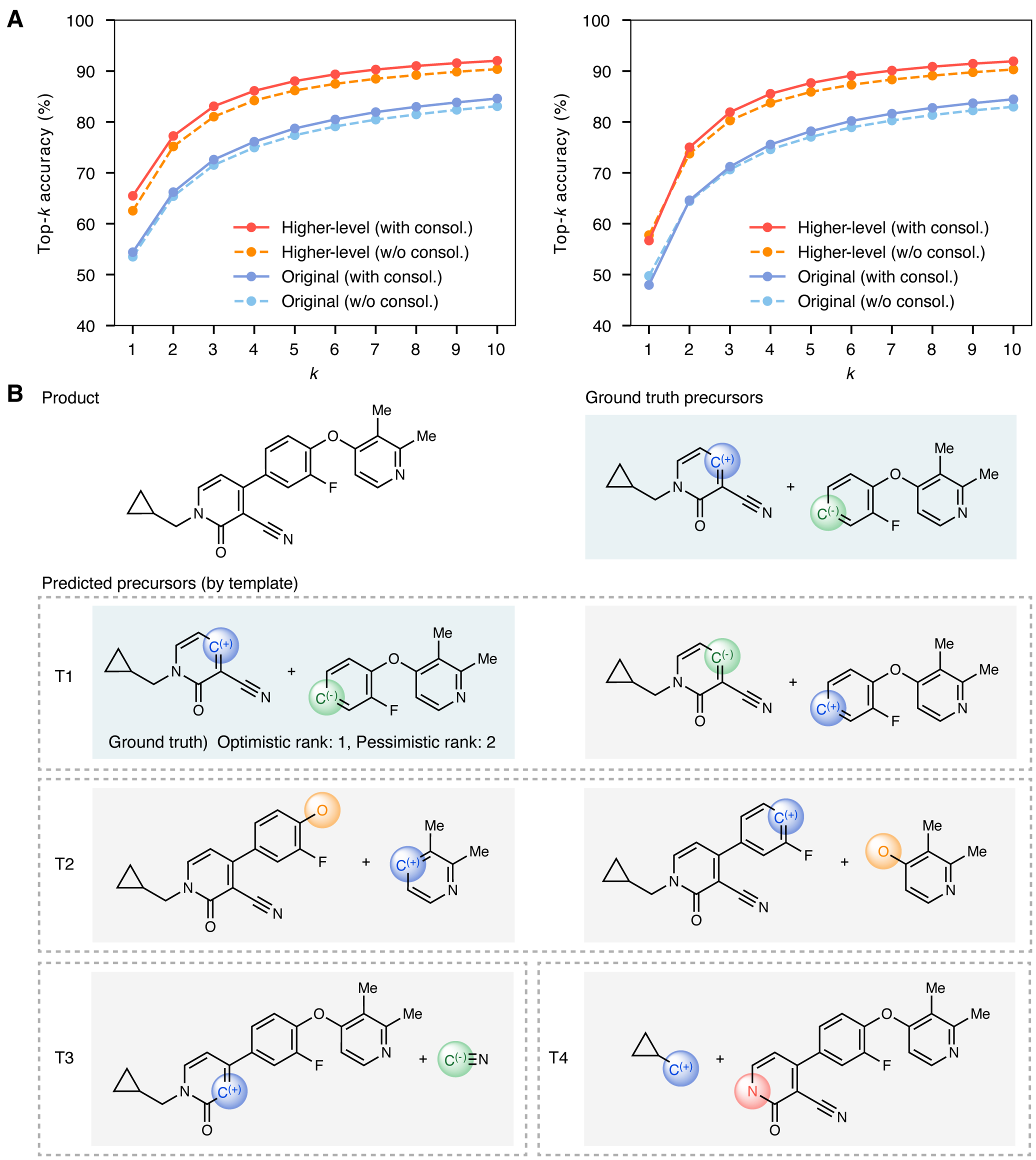

Figure S8: Single-step retrosynthesis model performance. (A) Top-k accuracy for higher-level and original models measured optimistically (left) and pessimistically (right), where the ground-truth is placed first and last, respectively, if a template gives multiple precursors. (B) Example prediction for molecule in the test set with the templates ranked in order. The ground truth precursors (blue) are generated by the highest ranking template (T1), which gives two different sets of precursors. The ranks for the ground truth are 1 (optimistic) and 2 (pessimistic).

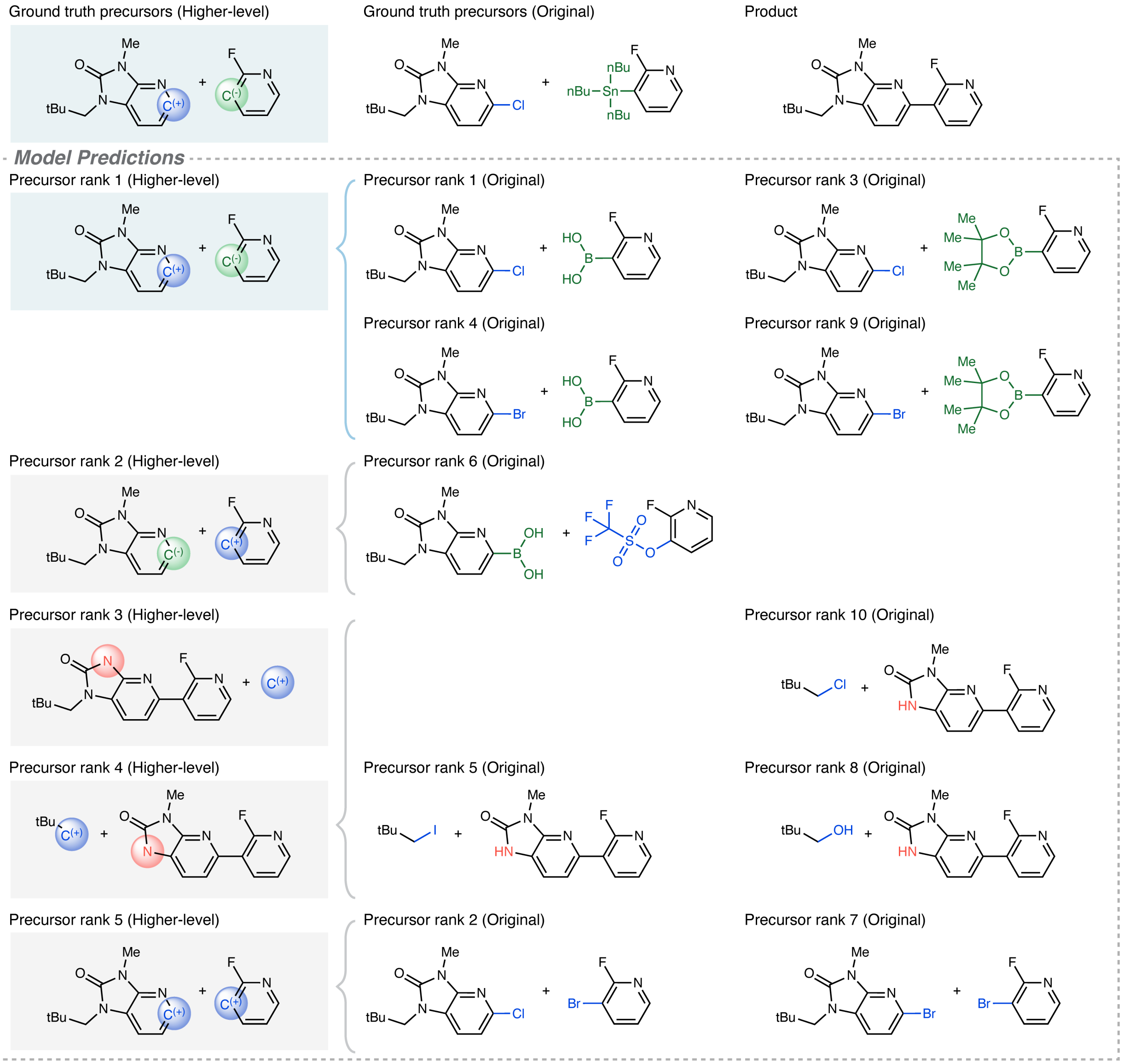

Figure S9: Comparison of single-step model results for the same input product molecule in the test set. The precursor ranks shown are both optimistic and pessimistic ranks, as all predicted templates generated exactly one set of precursors. The higher-level single-step model proposed the ground truth precursors as rank 1, while the original model failed to propose the ground truth precursors within the top 10 precursors. 4 out of the top 5 precursors by the higher-level model is able to represent the disconnections used in the top 10 precursors by the original model, demonstrating the higher-level model's capability to propose more diverse transformations. Additionally, the top-k accuracy in the higher-level space better reflects that the same strategy/transformation may involve multiple different potential functional groups, and thus proposed precursors could be *correct* even if they do not match the literature-recorded precursors, a limitation in the existing top-k exact-match accuracy (i.e., in the original space) that metrics like MaxFrag[12] have attempted to address.

S5.2 Developing the higher-level multistep retrosynthesis algorithm

For the higher-level algorithm, we expanded the Monte-Carlo tree search (MCTS) algorithm in ASKCOS,[13] which performs iterative steps of selection, expansion, and update.

S5.2.1 Details on selection, expansion, and update steps in MCTS

For multistep retrosynthesis, we use the Monte-Carlo tree search algorithm in ASKCOS.[13] Reactions and molecules are represented as nodes within the search graph, where each reaction node has a single molecule node as its parent (i.e., product of the reaction) and one or more molecule nodes as its children (i.e., reactant(s) of the reaction). The search tree is initiated with the input target molecule as the root node and the tree is expanded through iterative steps of selection, expansion, and update.

For selecting the next node to expand, we use a simplified Upper Confidence bound applied to Trees (UCT)[14] score. The UCT score for reaction node r with product M_p generated using template t is calculated as follows:

$$\text{Score} = Q_r + c * U_r \tag{1}$$

$$= \frac{\text{MLP}(t|M_p) * v_r}{n_r} + c * \sqrt{\frac{ln\,(N_{M_p})}{n_r}} \tag{2}$$

The UCT Score considers both exploitation (Q_r) and exploration (U_r), balancing them with the exploration weight c. The exploitation term Q_r considers the template probability predicted by the single-step model $\text{MLP}(t|M_p)$, the average buyability score (1.0 for buyables and 0.0 for non-buyables) of its children chemical nodes v_r, and the number of times the reaction node has been visited n_r. The exploration term U_r considers N_{M_p}, the number of times the parent product node has been visited, and n_r. Such a formulation enables the algorithm to explore less frequent but potentially significant transformations, ensuring that even rare but important reactions are given consideration, given that it is proposed by the single-step model. If a reaction with the highest UCT score has multiple non-buyable

reactants, the reactant node with the lowest visit count is selected. This process is repeated until an unexpanded leaf node is reached.

Once a leaf node has been selected, the single-step model is applied to the selected molecule to generate precursor predictions. The resulting precursor molecules are added as chemical nodes to the search tree, and reaction nodes connecting these precursors to the product molecule are also added. For each new chemical node, the buyability of the molecules is evaluated, and the average buyability scores and visit counts of nodes in the linear pathway from the root node of the search tree to the selected leaf node are updated. This process is repeated until there are no remaining nodes to expand within the given depth limit, or the iteration and/or time limit has been reached, and the resulting network is traversed to identify routes that end in terminal (i.e., buyable) nodes.

S5.2.2 Details on higher-level multistep retrosynthesis algorithm

The main modification from the original algorithm lies in how we evaluate the buyability of each abstracted molecule node. To determine whether each molecule node is buyable, complete molecules are cross-referenced against the buyables database using their SMILES strings. The buyability of abstracted molecule nodes in the higher-level algorithm is evaluated using substructure matching with SMILES arbitrary target specification (SMARTS) strings,[15] with the SMARTS strings constructed as the reverse of the abstraction heuristics. That is, $R_1{-}^a\mathrm{X}$ is allowed to match buyables with $R_1{-}X{-}Y{-}LG$ structure, where the atom allowed for Y and the bond between X and Y are determined by $^a\mathrm{X}$. For instance, $R_1{-}^5\mathrm{C}$ (i.e., $R_1{-}\mathrm{C}^{(-)}$) groups are only allowed to match buyable molecules with $R_1 - X - Y - LG$ structures if Y is a heavy atom less electronegative than C. Because this approach focuses on the identity of the connected atom (e.g., B in boronic esters), the algorithm is allowed to identify buyable molecules in the dataset that may not be present in the original USPTO reaction dataset, allowing for flexibility across various sets of buyables Here, we exclude matching buyables which require breaking a C−C single bond to make use of the starting material in the syntheses.

The buyable molecules are first filtered based on several criteria to decrease the number of substructure matching needed for each query: the maximum price per gram, the minimum number of rings, minimum number of heavy atoms, minimum number of C, N, O, F, P, S, Cl, and Br atoms, and the bits in the pattern fingerprint as implemented in RDKit.[10] The minimum number of rings and atoms correspond to the number of rings and atoms in the abstracted molecule, as they represent substructures of the molecule. The pattern fingerprint is a topological fingerprint that identifies whether the molecule includes a list of very generic substructures, which is optimized for substructure screening. Although not done in this work, substructure matching can be parallelized to further improve time efficiency in practical applications.

The buyable query also takes in the limit on the number of molecule matches to return,

which is a tunable hyperparameter. During the tree search, for computational efficiency, we set the limit to 1. Since the buyable molecules are sorted by the number of heavy atoms, the SMARTS pattern with the fewest number of heavy atoms is always returned during the search. This sorting and count limit is purely for computational efficiency and does not affect the definition of a successful match, which depends only on whether a molecule satisfies the SMARTS pattern. After the search has been completed, users can increase the limit and apply alternative sorting criteria (e.g., by size, cost, or predicted feasibility) to explore and prioritize additional available building blocks for downstream analysis. An example of ranking the matched buyable molecules using predicted feasibility scores is provided in Section S7.

S6 Evaluating the higher-level retrosynthesis algorithm

S6.1 Evaluation setup and parameters

We used the buyable building block database from ASKCOS for all pathway searches, which include 329,635 unique chemicals from vendors eMolecules, Sigma-Aldrich, Mcule, LabNetwork, and ChemBridge. The lowest price per gram information is used for chemicals that are available in multiple vendors. The parameters used for multistep experiments are summarized in Table S4 below.

Table S4: Parameters used for multistep experiments. The maximum expansion time is set arbitrarily high to allow the maximum number of iterations to be the dominant stopping criteria. All other filters or post-processing steps in the ASKCOS implementation of MCTS are turned off.

	**Parameter**	**Value**
**Single-step model parameters**	Max. number of templates	25
	Max. cum. probability	1.0
**Multistep search parameters**	Max. number of iterations	500
	Max. depth	8
	Max. branching	25
	Exploration weight	1
	Max. buyable ppg ($/gram)	100.0
	Max. expansion time (s)	12,000

S6.2 Benchmarking on USPTO-190 test molecules

The target molecules in the USPTO-190 dataset were used to provide a quantitative assessment of the performance of our algorithm. Once the search was completed, synthetic pathways were returned using a depth-first search in the final search tree. For each target molecule, we analyzed and compared the iteration at which the first pathway was identified, the minimum depth of the returned pathways, and the minimum number of reactions in the returned pathways. The performance of the higher-level and original algorithms, with and without template consolidation are summarized in Table S5 and Fig. S10.

Table S5: Multistep search results for USPTO-190 molecules. Number of solved molecules and success rate by pathway depth (d) and number of iterations (N).

		Higher-level		Original	
		with consol.	w/o consol.	with consol.	w/o consol.
Number of molecules solved (Success rate)[1]	$d \leq 1$	3 (1.58%)	2 (1.05%)	0 (0.00%)	1 (0.53%)
	$d \leq 2$	24 (12.6%)	17 (8.95%)	11 (5.79%)	7 (3.68%)
	$d \leq 3$	58 (30.5%)	41 (21.6%)	33 (17.4%)	25 (13.2%)
	$d \leq 4$	103 (54.2%)	83 (43.7%)	57 (30.0%)	46 (24.2%)
	$d \leq 5$	132 (69.5%)	113 (59.5%)	78 (41.1%)	60 (31.6%)
	$d \leq 6$	136 (71.6%)	120 (63.2%)	86 (45.3%)	71 (37.4%)
	$d \leq 7$	140 (73.7%)	123 (64.7%)	88 (46.3%)	73 (38.4%)
	$d \leq 8$	140 (73.7%)	124 (65.3%)	88 (46.3%)	73 (38.4%)
	$N \leq 5$	27 (14.2%)	23 (12.1%)	12 (6.32%)	9 (4.74%)
	$N \leq 10$	48 (25.3%)	34 (17.9%)	16 (8.42%)	12 (6.32%)
	$N \leq 50$	101 (53.2%)	95 (50.0%)	42 (22.1%)	40 (21.1%)
	$N \leq 100$	113 (59.5%)	114 (60.0%)	56 (29.5%)	54 (28.4%)
	$N \leq 300$	139 (73.2%)	123 (64.7%)	83 (43.7%)	68 (35.8%)
	$N \leq 500$	140 (73.7%)	124 (65.3%)	88 (46.3%)	73 (38.4%)
Number of molecules solved by each[2]		140	124	88	73
Number of iterations for first path[2]		45.7 ± 62.9	39.8 ± 51.9	93.2 ± 97.5	85.4 ± 108.6
Minimum pathway depth[2]		3.74 ± 1.24	3.98 ± 1.28	3.99 ± 1.25	4.12 ± 1.31
Minimum number of reactions[2]		4.19 ± 1.59	4.45 ± 1.58	4.30 ± 1.52	4.47 ± 1.61
Number of molecules solved by all[3]		68			
Number of iterations for first path[3]		$\mathbf{18.7 \pm 27.9}$	21.7 ± 29.2	73.8 ± 79.5	82.8 ± 107.8
Minimum pathway depth[3]		$\mathbf{3.10 \pm 1.00}$	3.54 ± 1.17	3.87 ± 1.16	4.10 ± 1.31
Minimum number of reactions[3]		$\mathbf{3.26 \pm 1.16}$	3.74 ± 1.28	4.24 ± 1.51	4.47 ± 1.62

[1] Maximum depth and maximum number of iterations is each kept at 8 and 500 during network exploration. The explored network is pruned once the exploration is complete to enumerate pathways at each limit and find the success rate. $N \leq 500$ for varying d limits and $d \leq 8$ for varying N limits. Note that d is the depth of individual pathways, which differs from the depth of the entire network.
[2] Values are calculated for the molecules that are solved by each model/algorithm.
[3] Values are calculated for the molecules that are solved by all models/algorithms (i.e., 68 molecules).

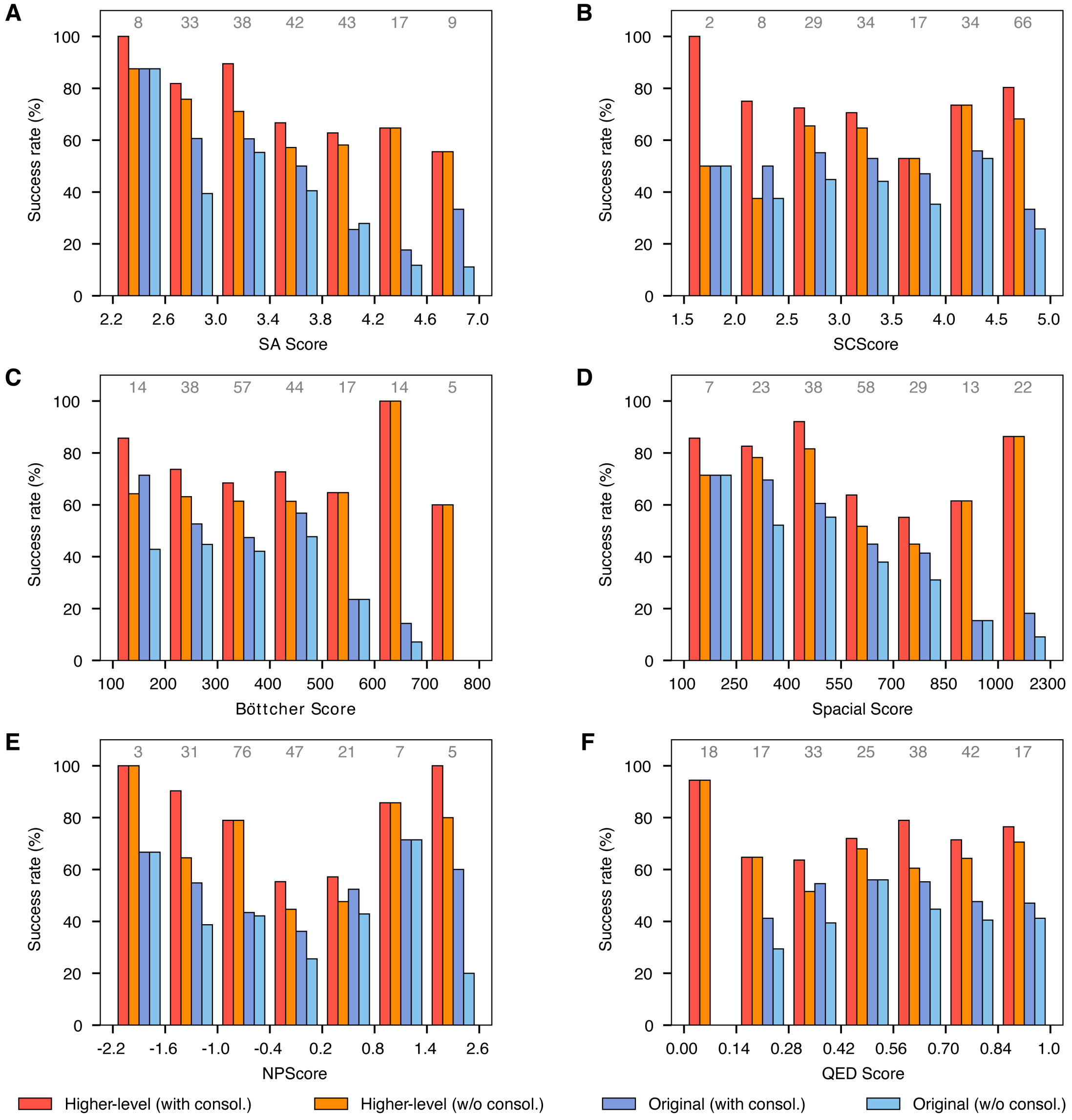

Figure S10: Multistep search success rate for the target molecules in USPTO-190 dataset for higher-level and original algorithms with and without template consolidation. Success rate are measured by synthetic complexity metrics: (A) SA Score[16] and (B) SCScore,[17] structural complexity metrics: (C) Böttcher Score[18] and (D) Spacial Score,[19] (E) natural-product likeness score (NPScore),[20] and (F) quantitative estimates of drug-likeness (QED) score.[21] Higher scores correspond to higher complexity or natural-product/drug likeness. The number of molecules in each bin are indicated in gray. SA Score, NPScore, and QED score were computed as implemented in RDKit.[10] SCScore, Böttcher Score, and Spacial Score were computed using the SCScore and molecular complexity modules in ASKCOS.[13]

S6.2.1 Contextualization relative to multistep retrosynthesis algorithms with value functions

Several prior works have reported strong multistep retrosynthesis performance on the USPTO-190 benchmark using value-guided search algorithms such as Retro*[7] and Planning with Dual Value Networks (PDVN).[22] While a direct quantitative comparison is not meaningful due to differences in reaction preprocessing, single-step models, and definitions of available building blocks, which strongly influence benchmark performance,[23,24] we briefly contextualize the present framework relative to these approaches.

Retro* and PDVN formulate multistep retrosynthesis as a tree or graph search problem guided by learned value functions that estimate the synthetic cost or feasibility of intermediate nodes, which are fully specified molecules. When coupled with strong single-step models, these approaches achieve highly competitive performance on datasets such as USPTO-190, where search is conducted over complete molecular structures.

Our work focuses on a higher-level retrosynthesis formulation in which molecules are abstracted to emphasize strategic disconnections rather than precise functional group choices. Under this formulation, intermediate nodes no longer correspond to fully specified molecules, and therefore value functions trained to assess the feasibility or cost of molecules cannot be directly applied without substantial adaptation. As a result, any value guided search method would require a principled strategy for evaluating abstracted intermediates.

In this work, we adopt MCTS without a rollout phase as a simple and flexible search strategy that can operate directly on abstracted representations without requiring a separate value function over intermediate nodes, which allows us to isolate and study the impact of higher-level abstraction on multistep planning behavior. However, the present framework is not inherently limited to MCTS. Once reliable methods for assessing the synthesizability or feasibility of intermediate nodes in the higher-level setting are developed, value guided approaches such as Retro* or PDVN could be naturally incorporated. We therefore view these methods as promising and complementary extensions of the current framework.

S7 Evaluating feasibility of matched buyable molecules

Feasibility is an essential consideration in synthesis planning. Assessing the chemical feasibility of predicted reactions and synthetic routes remains a significant challenge in computer-aided synthesis planning. Typically, the question of whether a suggested retrosynthetic pathway is feasible is equivalent to the question of whether there is any set of conditions that would successfully produce the desired product in non-negligible yield. As our approach focuses on abstract representations of molecules, feasibility assessment becomes more difficult, as it broadens that question to whether there is any set of conditions for any tactical implementation/variation on the specified strategy that would successfully produce the desired product in non-negligible yield. This potentially leads to optimistic success estimates, particularly in cases where the matched building blocks or resulting intermediates cannot successfully execute the proposed strategy even with reasonable modifications. To begin to address the potential concern of feasibility in our framework, we propose the incorporation of forward reaction predictor models, which predict the likely products for a given set of reactants.[25] These models can serve as a proxy for reaction feasibility, although their predictions are not intended to serve as definitive measures. While a complete integration of feasibility assessment is beyond the scope of this work, we explore how such tools might be incorporated into our framework through a targeted example.

We use the higher-level route proposed in Fig. 4A as an example, focusing on the transformation between intermediates **12** and **13** (Fig. S11). Specifically, we evaluate whether the matched building blocks identified for this step are chemically plausible participants in the proposed transformation. We use the forward predictor in ASKCOS (wldn5 model, trained on Pistachio dataset)[13] to predict likely reaction outcomes for different combinations of matched building blocks. To assess feasibility, we check whether the product formed by applying the intended transformation to the selected building blocks appears among the predicted outcomes. The probability assigned to this product by the model is then used as a proxy for reaction feasibility. In cases where the starting materials contain abstract groups

that are not directly involved in the transformation, the functional groups can be propagated from the matched building blocks to the corresponding positions in the product.

The assigned feasibility scores can be used to rank the matched building blocks or to filter out those with low predicted feasibility. This enables a flexible post-processing step that complements our framework's focus on higher-level disconnections.

This analysis is not intended as a comprehensive feasibility evaluation. Our framework is explicitly designed to focus on strategic-level disconnections rather than specific functional group choices or the modifications required to interconvert between them. As such, a matched building block may sometimes require additional adjustment, such as functional group interconversions (FGIs), to participate in the intended transformation. This is a deliberate aspect of the framework's design, where the tactical considerations are deferred in order to emphasize higher-level retrosynthetic planning. That is, tactical steps (e.g., Fig. 2B, step **ii**) were removed during dataset curation to isolate strategic disconnection logic. Consequently, the matched building blocks may need these additional steps in the forward direction to successfully participate in the proposed transformation. Because the forward predictor only accounts for cases where the matched building block directly participates in the proposed transformation, a low model score or the absence of the product from the predictions does not necessarily imply that the route with the matched building blocks is infeasible.

However, we note that due to the generalized form of the starting materials in our approach, there may be cases where none of the matched building blocks is suitable, even with modifications to them in the forward direction. In these instances, additional disconnections or transformations in the retrosynthetic direction may be required to access suitable starting materials. For example, in Fig. 4A, the original algorithm proposes a nucleophilic attack of isocyanate **15** by amine **16** to form substituted urea **14**. In contrast, the higher-level algorithm treats **10**, the higher-level equivalent of **14**, as an acceptable starting material, as it identifies a structurally matching buyable molecule, which may or may not be suitable as a starting material. To better account for these cases, the stopping criterion of the algorithm

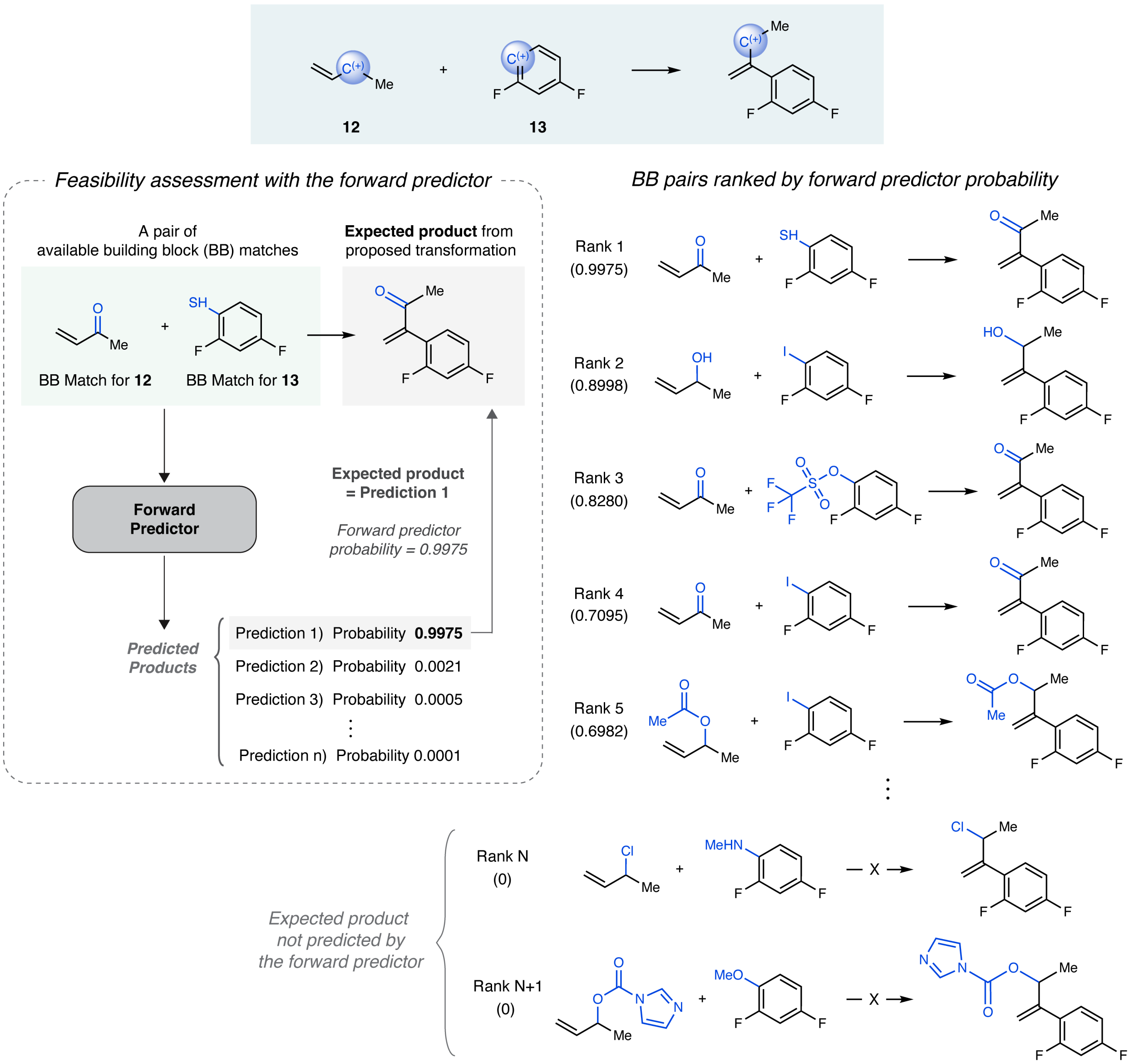

Figure S11: Scoring and ranking of matched building blocks (BBs) by estimated reaction feasibility. In this example, a pair of buyable building blocks matched with higher-level starting materials **12** and **13** are given to the forward predictor in ASKCOS. The forward predictor is used to predict likely products, and the model probability of the expected product, if it exists in the predictions, is assigned as the score for this pair of building blocks (left). Then, all possible combinations of the building blocks are fed to the forward predictor in the same way, and the building blocks can be ranked by combination required for the step (right), or individually (e.g., maximum probability for the specific building block). Note that there could be cases where the forward predictor does not give the expected product within the top-n predictions, which can be an indication of low perceived feasibility.

could be relaxed to allow further expansions of nodes with building block matches. This could, for example, allow **10** to be further disconnected into higher-level equivalents of **15** and **16**, offering alternative starting points that may be more promising in practice. While this extension is not implemented in the current work, it represents a promising direction for improving route quality and diversity in future versions of the algorithm.

S8 Case studies with drugs and natural products

The case studies present a qualitative evaluation to illustrate the behavior of the proposed framework in practical settings, rather than to provide a systematic benchmark. Using the setup in Section S6.1, we conducted automatic searches using drug molecules and natural products with known synthesis pathways from Nicolaou and Sorensen[26] and the Chemistry By Design (CByD) database by the Njardarson group.[27] Literature searches were conducted to map the higher-level routes back to retrosyntheses with complete molecular structures. The templates associated with each of the steps in the higher-level route and their literature precedents were used as a guide in this process (Figs. S16 and S17). Chemical intuition was used to compare the suggested disconnections with those done in the lab. The flexibility of the model's suggestions was used to merge chemists' recommendations with the model's retrosynthetic route, thus enabling strategic routes to be proposed.

S8.1 Overview of case-study results

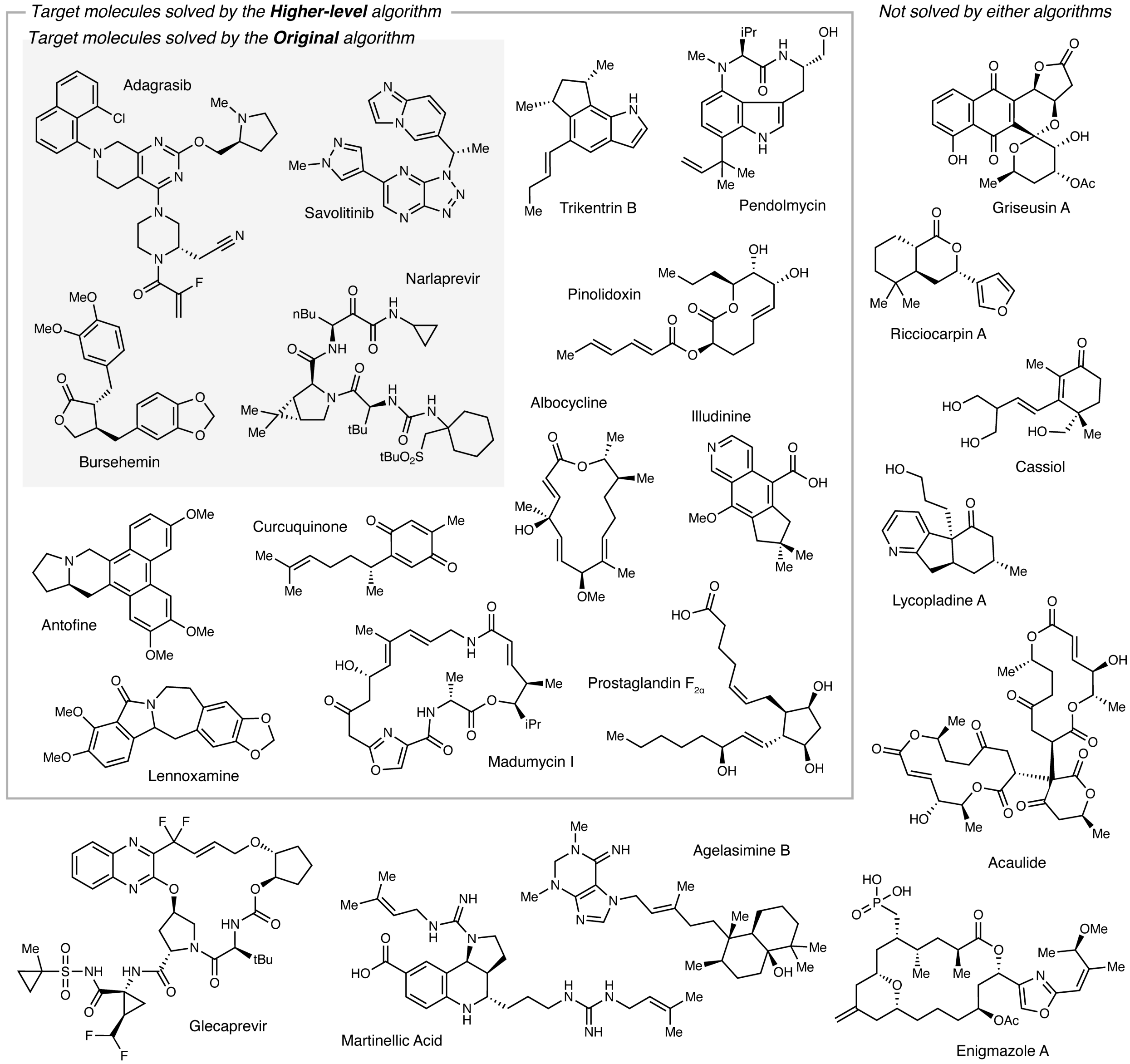

Figure S12: Representative examples of drugs and natural products used as target molecules used in the automated multistep tree search. Molecules are grouped depending on whether they are solved by both the higher-level and original algorithms (gray box), solved the higher-level algorithm only (gray outline), or solved by neither algorithms.

Fig. S12 illustrate examples of natural products and drug molecules used in the case studies. The higher-level algorithm successfully proposes routes for all targets solved by the original algorithm, as well as additional ones the original method fails to propose routes for.

Failures of the original approach arise from several compounding factors. A common

factor is that the single-step model proposes multiple reactions involving the same general strategy but applied to different functional groups (e.g., variations in leaving groups), which limits the diversity of disconnections explored within search parameters that restrict the search width, such as branching ratio and the number of templates applied (Fig. S9), and can lead to repeated application of similar templates to closely related molecules differing only in minor functional group modifications across iterations. Additionally, the algorithm suggests sequences of functional group interconversions or protecting group manipulations, which consume the available search depth without making meaningful progress toward complexity reducing disconnections.

Additional failure modes for both approaches can arise from the scope of the training data and search parameters, including branching ratio, maximum number of templates applied, maximum search depth, and the list of buyable building blocks, all of which significantly affect performance. We later demonstrate in Section S9 that expanding training set diversity and adjusting these parameters enables the higher-level algorithm to propose routes for target molecules that were not successfully solved in this section. While these factors impact both methods, they are more restrictive for original algorithm due to the specific failure modes described above.

S8.2 Additional details on main-text case studies

This section includes additional details on case studies involving target molecules featured in the main text.

S8.2.1 Case study: Pinolidoxin

Additional details on the synthesis proposed based on the higher-level route in Fig. 6

From the retrosynthetic outline in Fig. 6A, we developed a full forward synthesis depicted in Fig. 6B. Here, we provide the details of the proposed transformations. To synthesize (–)-pinolidoxin, hemiacetal **42** – a readily available derivative of ribofuranose – could undergo acid-mediated ring opening and oxidation of the primary alcohol to give dialdehyde **43**.[28] A desymmetrizing organometallic addition of an *N*-propyl nucleophile, catalyzed by a titanium Lewis acid, could give rise to secondary alcohol **45** in a diastereoselective fashion, forging the first of two coupling partners necessary for the synthesis of (–)-pinolidoxin. As an alternative to this potentially-problematic asymmetric addition step, **45** could arise from protection and reductive opening of commercially-available D-lyxono-1,4-lactone (**44**).[29] Carboxylic acid **50** could arise from primary amine protection, Katritzky salt formation, and pyridinium displacement of commercially available D-ornithine **46** with sorbic acid (**49**). A Mitsunobu reaction[30] between secondary alcohol **45** and carboxylic acid **50** would give rise to aldehyde **51** after amine deprotection. Because the pendant amine of **51** would likely condense onto the aldehyde motif, the aldehyde could be protected prior to amine deprotection. Oxidation of the primary amine of **51** to an aldehyde by formal dehydrogenation and hydrolysis mediated by TEMPO and PIDA[31] would give rise to dialdehyde **52**, which is poised for a reductive McMurry coupling[32] to forge the oxecanone ring of the natural product. A deprotection of the vicinal diol will give (–)-pinolidoxin (**34**). Note that esters have higher reduction potential than aldehydes, and thus would typically require more forcing conditions than aldehydes to participate in McMurry coupling.[33]

Another proposed synthesis for the same higher-level route in Fig. 6

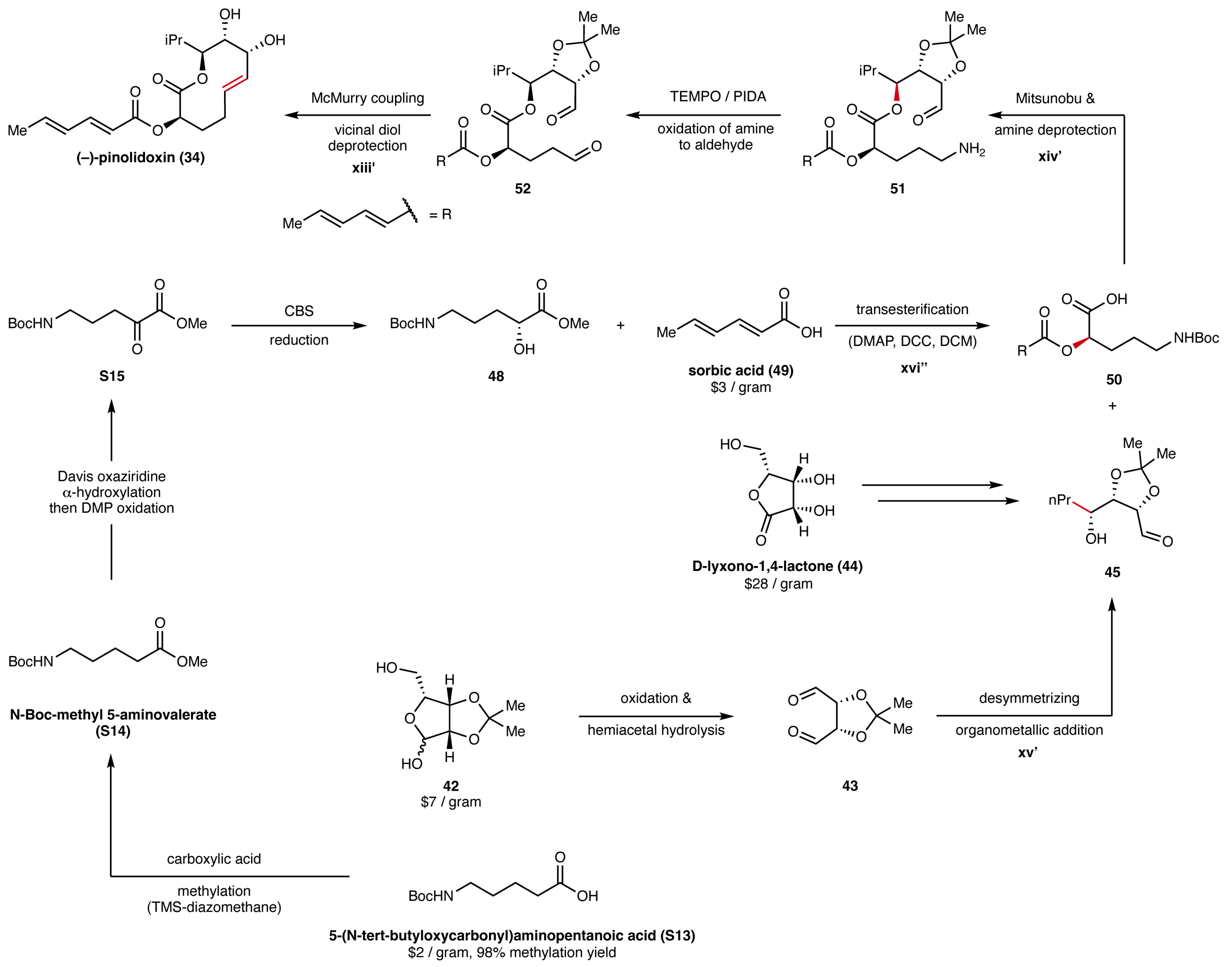

Figure S13: Case study: Pinolidoxin 2. A second forward synthetic route for (–)-pinolidoxin, constricted using standard transformations. The steps corresponding to steps $\boldsymbol{r_i}$ in the higher-level route (Fig. 6A) labeled as $\boldsymbol{r'_i}$ or $\boldsymbol{r''_i}$. The disconnected bonds in these steps are highlighted in red.

In addition to the synthetic route for (–)-pinolidoxin in Fig. 6B, we construct a different route based on the same proposed higher-level route in Fig. 6A using standard transformations with well-established, robust reaction conditions (Fig. S13). This proposed synthetic route for (–)-pindolidoxin (**34**) begins with a McMurry coupling[32] of dialdehyde **52**, and acetonide hydrolysis. Preceding this step, the pendant primary amine of **51** is oxidized to an aldehyde using TEMPO and PIDA.[31] Aldehyde **51** is obtained through a Mitsunobu reaction[30] between carboxylic acid **50** and secondary alcohol **45**,[30] followed by

amine deprotection of intermediate **50**. Carboxylic acid **50** is constructed *via* transesterification between sorbic acid (**49**, \$3/gram) and the *N*-Boc-protected amine **48**. To access **48**, CBS reduction[34] of ester **S15** yields the corresponding α-hydroxy ester. The preparation of **S15** involves Davis oxaziridine-mediated α-hydroxylation[35] of *N*-Boc-protected methyl ester **S14**, followed by oxidation to the ketone with DMP.[36] *N*-Boc-methyl 5-aminovalerate (**S14**) arises from precedented, TMS-diazomethane-mediated methylation[37] of carboxylic acid (**S13**, \$2/gram).[38] Parallel to this sequence to prepare **50**, secondary alcohol **45** arises from a desymmetrizing organometallic addition of an n-propyl nucleophilie into **43**, itself prepared from oxidation and hemiacetal hydrolysis of protected ribofuranose **42**.

Additional examples higher-level routes proposed for (–)-pinolidoxin

Alternative ***higher-level*** *route 1*

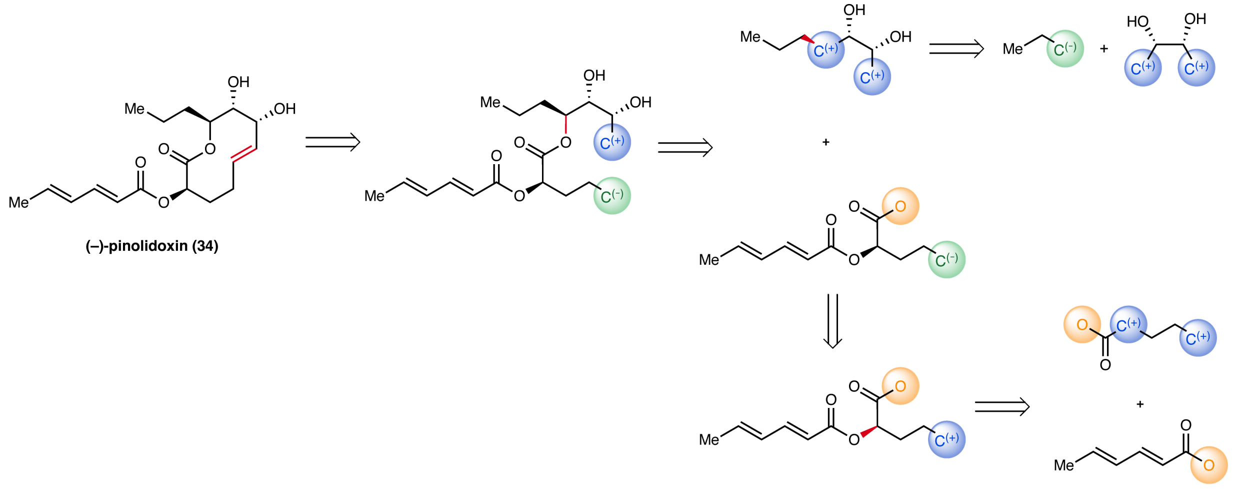

Alternative ***higher-level*** *route 2*

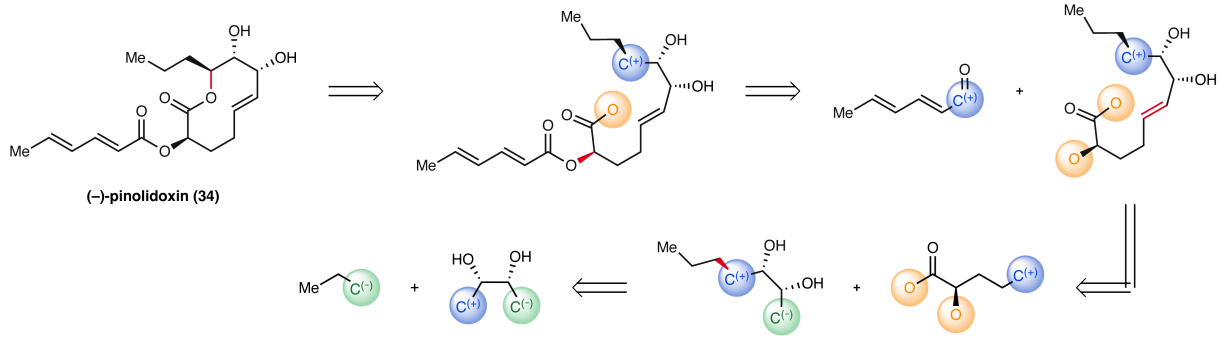

Figure S14: Case study: Pinolidoxin - Alternative higher-level routes. Two additional higher-level retrosynthetic routes for (–)-pinolidoxin identified by our framework. Alternative higher-level route 1 was selected to be similar to the route in Fig. 6A, sharing most key disconnections with one or two variations. Alternative higher-level route 2 was chosen to illustrate a route with more distinct strategies, differing in several core transformations. The disconnected bonds in these steps are highlighted in red.

S8.2.2 Case study: Pendolmycin

Additional details on the synthesis proposed based on the higher-level route in Fig. 7

Based on the proposed higher-level route for (–)-pendolmycin (**53**) in Fig. 7A, we propose the forward synthesis in Fig. 7B. This synthesis commences with an intramolecular Buchwald-Hartwig cross-coupling of compound **73**, engaging its methylamine and aryl halide (or pseudohalide) groups to forge the critical 9-membered macrocycle. Preceding this, peptide coupling of the primary amine of **71** with commercially available Boc-*N*-Me-L-Val-OH (**72**, \$2/gram, enantioenriched) furnishes compound **73**. The synthesis of **71** involves site-selective C5-halogenation of **70**, followed by ester reduction to yield a pendant primary alcohol and amine deacetylation under standard MeOH/K_2CO_3 conditions. The assembly of **70** is achieved through an enantioselective Michael addition of indole **69** to methyl 2-acetamidoacrylate (**68**, \$6/gram), leveraging a 2021 methodology from the Reisman group for asymmetric indole functionalization.[39] Indole **69** itself arises from the deacetylation of *N*-acylindole **67**. Two pathways are proposed to access **67**: a directed C–H allylation of *N*-acylindole **64** with prenyl alcohol (**65**, \$3/gram) to generate reverse-prenylated **67** or, alternatively, a C7-selective C–H borylation of **64**, followed by a cross-coupling to install the prenyl group.

Additional examples higher-level routes proposed for (–)-pendolmycin

*Alternative **higher-level** route 1*

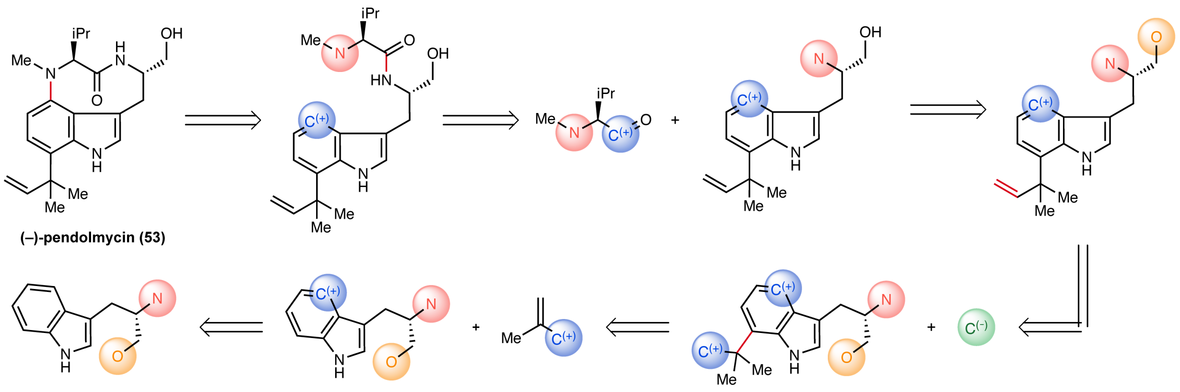

*Alternative **higher-level** route 2*

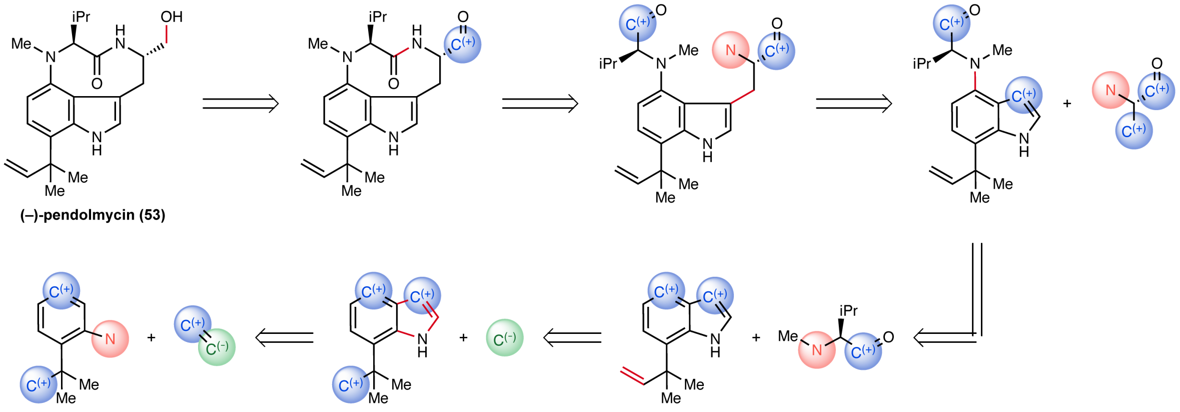

Figure S15: Case study: Pendolmycin - Alternative higher-level routes. Two additional higher-level retrosynthetic routes for (–)-pendolmycin identified by our framework. Alternative higher-level route 1 was selected to be similar to the route in Fig. 7A, sharing most key disconnections with one or two variations. Alternative higher-level route 2 was chosen to illustrate a route with more distinct strategies, differing in several core transformations. The disconnected bonds in these steps are highlighted in red. The disconnected bonds in these steps are highlighted in red.

S8.3 Additional Case Studies

We include examples of routes proposed by the higher-level algorithm for three additional natural products in which only the higher-level algorithm is able to propose potential routes for. One notable example is for illudinine, a sesquiterpene natural product found in *Omphalotus olearius* and *Omphalotus illudens* that inhibits monoamine oxidase B.[40,41] The higher-level algorithm proposed multiple potential routes, with the first pathway being identified at iteration 46 (Fig. S16). Another example is for albocycline, a macroline natural product isolated from *Streptomyces maizeus* with antibiotic activity,[42,43] where the higher-level algorithm is able to identify the first pathway at iteration 438 (Fig. S17). Finally, we include an example route for madumycin I, a streptogramin antibiotic,[44,45] with the first route identified by the higher-level algorithm at iteration 458 (Fig. S18).

S8.3.1 Additional case study: Illudinine

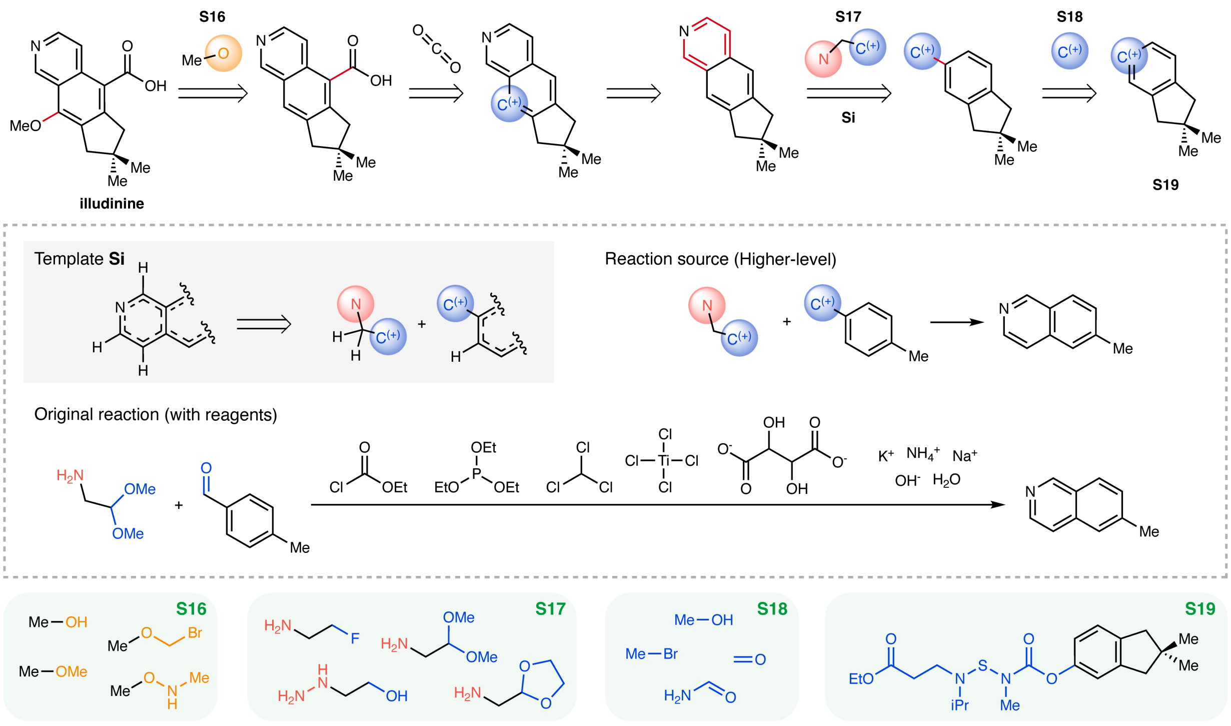

Figure S16: Case study: Illudinine. One of the shortest pathways with the fewest number of reactions proposed by the higher-level algorithm for illudinine is shown, with the disconnected/changed bonds highlighted in red. The retrosynthetic template, a source higher-level reaction, and its original reaction with reagents for proposed step **Si** are shown in the gray box. Example buyable molecules for starting materials **S16-S19** in the pathway are shown in green boxes.

S8.3.2 Additional case study: Albocycline

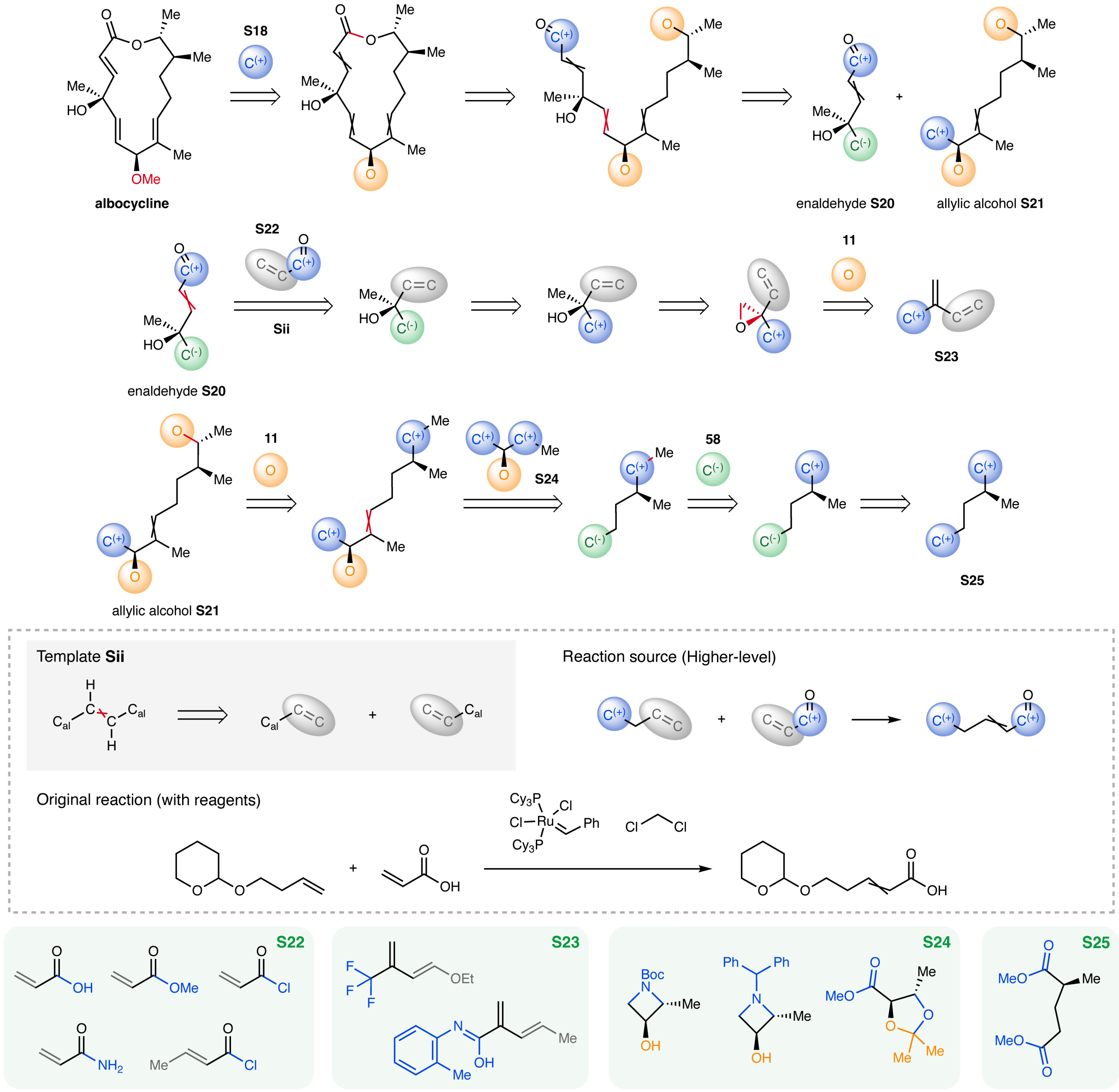

Figure S17: Case study: Albocycline. One of the shortest pathways with the fewest number of reactions proposed by the higher-level algorithm for albocycline is shown. The disconnected/changed bonds at each step is highlighted in red. The retrosynthetic template, a source higher-level reaction, and its original reaction with reagents for proposed step **Sii** are shown in the gray box. Example buyable molecules for starting materials **S22-S25** in the pathway are shown in green boxes. See Figs. 4, 7, and S16 for example buyable molecules for **11**, **58**, and **S18**, respectively.

S8.3.3 Additional case study: Madumycin I

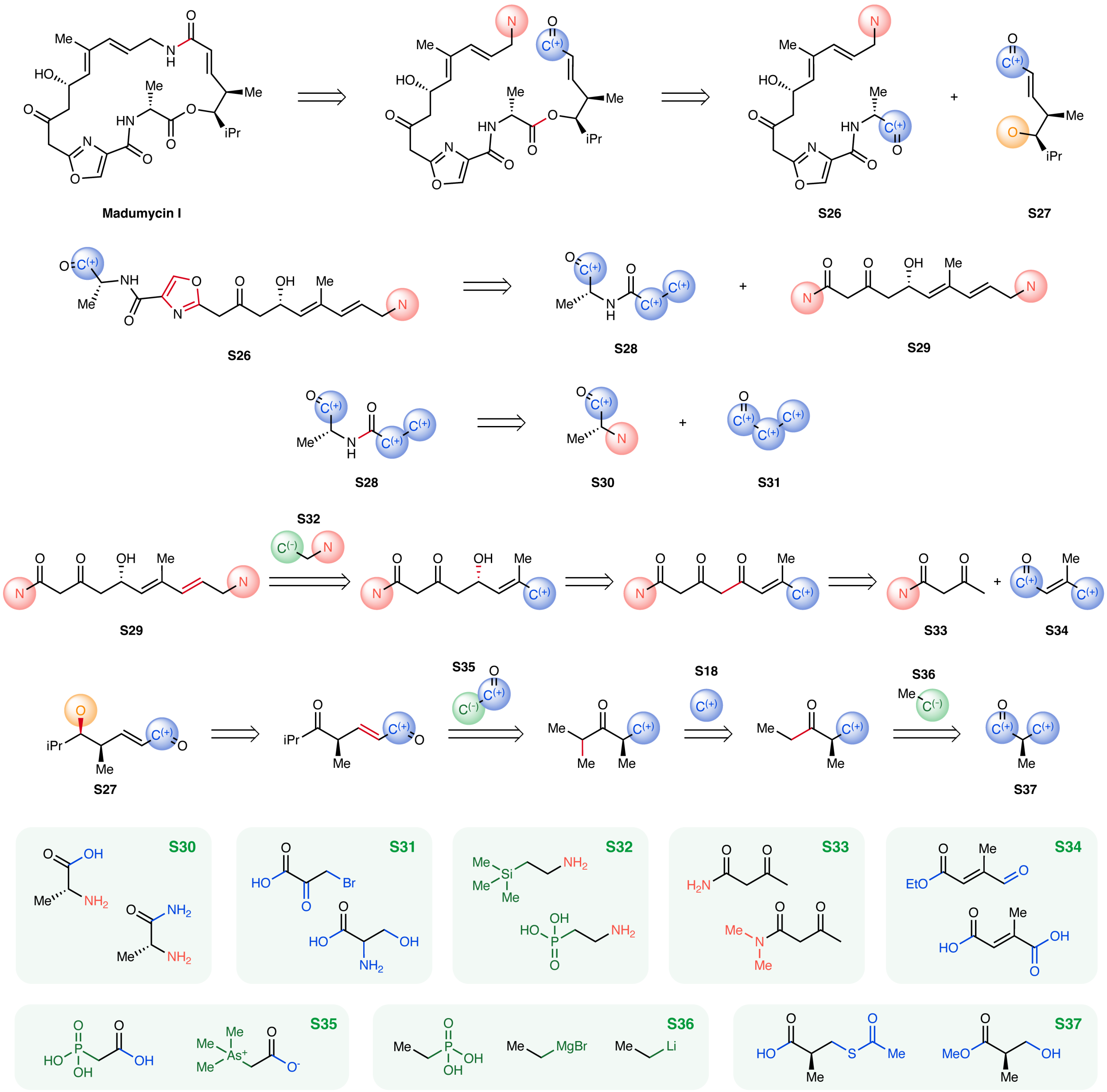

Figure S18: Case study: Madumycin I. One of the shortest pathways with the fewest number of reactions proposed by the higher-level algorithm for madumycin I is shown. The disconnected/changed bonds at each step is highlighted in red. Example buyable molecules for starting materials **S30-37** in the pathway are shown in green boxes. See S16 for example buyable molecules for **S18**.

S9 Generalizing to the larger, more diverse Pistachio dataset

We used the USPTO dataset for all main-text results and all other supplementary sections (except for this one) because it is open-source, widely used in benchmarking, and enables full reproducibility of both model training and search outputs. However, USPTO is known to be relatively limited in reaction diversity. To evaluate the comprehensiveness and generalizability of our approach, we applied our methods to the Pistachio dataset (2024Q4 version)[2] to generate a larger, more diverse higher-level route and reaction datasets. We trained single-step models using this dataset, with the results presented in Section S9.1. To further assess the impact of training data diversity on multistep route generation, we also conducted case studies with the higher-level retrosynthesis algorithm using the Pistachio-trained model with expanded search parameters (Section S9.2). These searches produced broader and more varied retrosynthetic routes, including strategies not accessible with the higher-level algorithm using the USPTO-trained model in Section S8.

S9.1 Template extraction, single-step model training and performance

We followed the same procedure with the Pistachio dataset as we did as for the USPTO dataset to train both the higher-level and original models, with one modification in the template consolidation strategy. Due to the significantly larger number of reactions and extracted templates in the Pistachio dataset, it was computationally prohibitive to exhaustively apply all templates to all reactions during template consolidation. As a result, we implemented a relaxed consolidation strategy: extracted templates were first sorted by frequency (i.e., number of literature precedents), from most to least common, and each reaction was matched to the first template in this ordered list that successfully recovered the recorded reactants. The resulting higher-level and original reaction datasets contained 3,827,669 and 3,486,548 deduplicated reactions, respectively. The train/validation/test splits included 3,062,135/382,766/382,768 reactions for the higher-level dataset and 2,789,238/348,654/348,656 reactions for the original dataset.

The diversity of the chemistry in the Pistachio dataset are reflected in the number of consolidated templates. That is, the higher-level dataset gave 105,684 templates, and the original gave 176,133 (Table S6)), compared to 32,141 and 51,736 templates, respectively, in the USPTO dataset (Table S3.

Our approach scales effectively, with the higher-level model (with template consolidation) recovering 92.82% of ground-truth strategies in the top-10 predictions even when applied to the much larger and more diverse Pistachio dataset (Table S7).

Table S6: Hyperparameters used to train each single-step model with the Pistachio dataset.

	**Higher-level**		**Original**	
	with consol.	w/o consol.	with consol.	w/o consol.
Dropout	0.3	0.1	0.1	0.4
Learning Rate ($\times 10^{-3}$)	0.55585	0.64001	0.80201	0.58388
Hidden Activation	ReLU	ReLU	ReLU	ELU
Number of Hidden Layers	1	1	1	1
Hidden Sizes	2,048	2,048	1,024	1,024
Input Dimension	2,048	2,048	2,048	2,048
Output Dimension[1]	106,106	370,193	176,133	418,847

[1]Output dimension is equal to the number of templates.

Table S7: Top-k accuracy (%) for each single-step model trained on the Pistachio dataset.

		**Higher-level**		**Original**	
Ranking type	k	with consol.	w/o consol.	with consol.	w/o consol.
Optimistic	1	63.94	61.57	51.77	51.09
	3	83.41	81.44	71.62	71.10
	5	88.72	86.76	78.26	77.50
	10	92.82	91.18	84.53	83.78
Pessimistic	1	55.01	56.37	45.40	47.08
	3	81.73	80.30	69.91	70.01
	5	88.08	86.29	77.48	76.92
	10	92.66	91.06	84.30	83.59

S9.2 Case studies with Pistachio models

We conducted additional case studies using the higher-level algorithm with the model trained on the Pistachio dataset (with template consolidation). For these experiments, we adjusted the parameters in Table S4 to allow for deeper and broader exploration, as summarized in Table S8.

Table S8: Parameters used for expanded multistep experiments. All filters or post-processing steps in the ASKCOS implementation of MCTS are turned off unless specified in the table below.

	**Parameter**	**Value**
**Single-step model parameters**	Max. number of templates	50
	Max. cum. probability	1.0
**Multistep search parameters**	Max. number of iterations	1000
	Max. depth	12
	Max. branching	50
	Exploration weight	1
	Max. buyable ppg ($/gram)	100.0
	Max. expansion time (s)	60,000

S9.2.1 New routes to previously solvable targets

The higher-level algorithm with the higher-level Pistachio model proposed more routes for targets that had already been solved using the higher-level USPTO model (Section S8). These alternative routes involve distinct disconnection strategies and/or reduced pathway depth. Representative examples are shown in Figure S19.

*Alternative **higher-level** route for (–)-pinolidoxin (Pistachio model)*

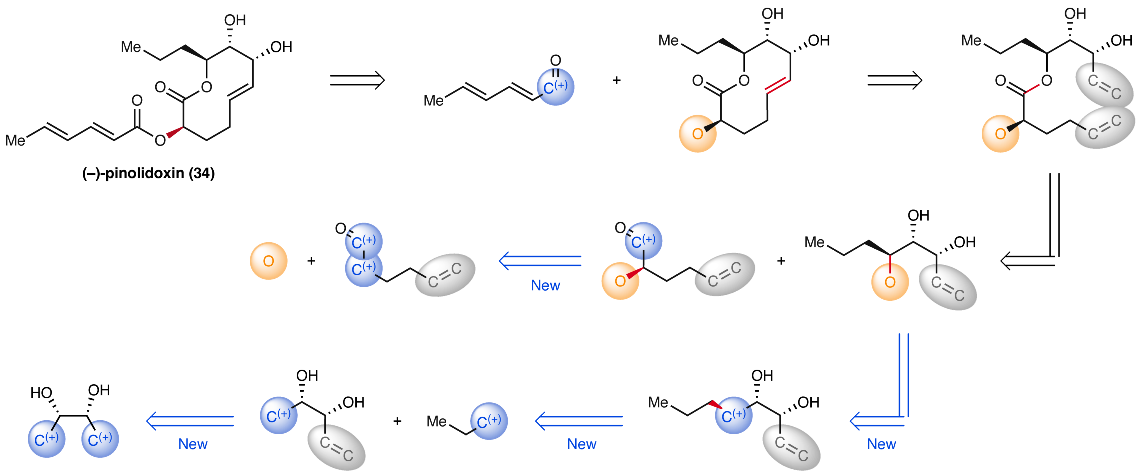

*Alternative **higher-level** route for (–)-pendolmycin (Pistachio model)*

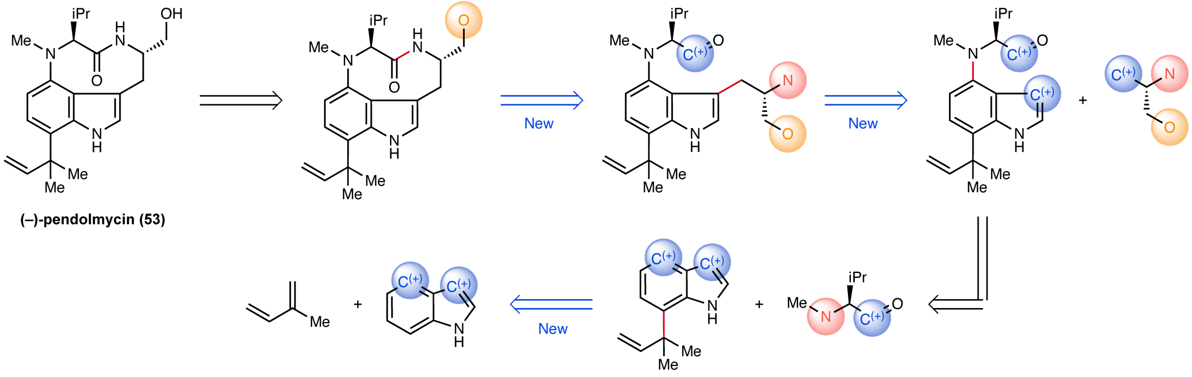

Figure S19: Case studies: Pinolidoxin and Pendolmycin - Higher-level routes proposed using the pistachio model. Steps highlighted in blue indicate those unseen during the case study in Section S8. The disconnected/changed bonds at each step are highlighted in red.

S9.2.2 Additional solvable targets

In Fig.S20, we present a representative example of a target molecule, glecaprevir, for which the higher-level algorithm with the Pistachio model successfully proposed routes. This target was not solved using the higher-level USPTO model in the case study in Section S8.

*Proposed **higher-level** route for glecaprevir (Pistachio model)*

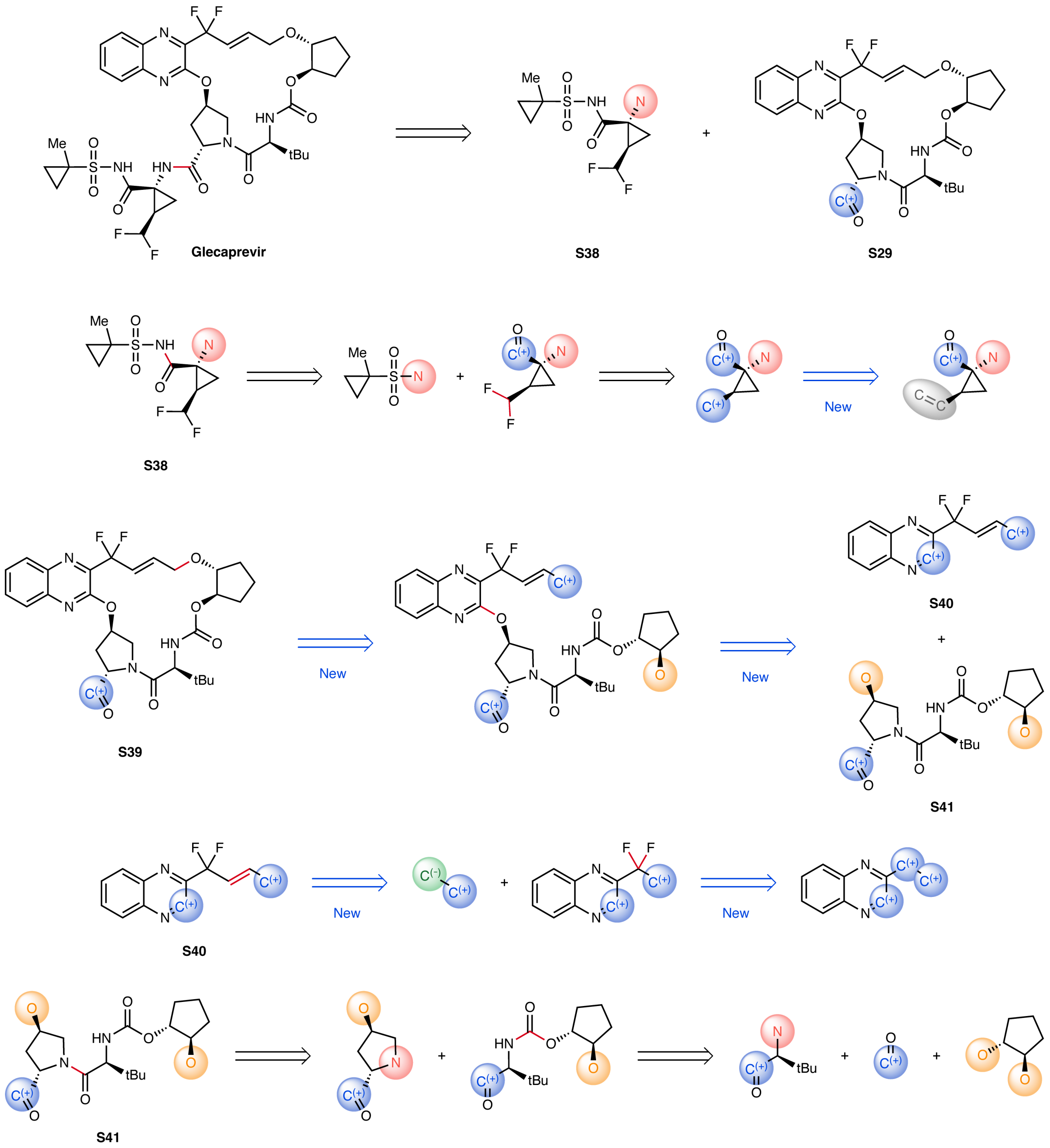

Figure S20: Higher-level routes proposed using the Pistachio model for glecaprevir. The higher-level retrosynthesis algorithm with the higher-level Pistachio model successfully proposed routes for glecaprevir, a target that was not solved using the USPTO model in Section S8. Steps highlighted in blue were not observed in the USPTO-model based experiment. The disconnected/changed bonds at each step are highlighted in red.

S10 Comments on the role of experimental validation

Experimental validation plays a central role in chemical research and has been an important component of many prior computer-aided synthesis planning (CASP) studies. In particular, experimental demonstrations have been used to establish that algorithmically generated synthesis plans can be translated into laboratory execution. Notably, the Synthia program developed by Grzybowski and co-workers was used to design synthetic pathways for eight structurally diverse targets, which were successfully executed in the laboratory after condition selection, convincingly demonstrating the practical value of CASP in delivering executable synthetic routes.[46] Later work demonstrated experimental validation of an even more challenging set of targets.[47] Similarly, machine learning-based synthesis planning of simpler structures has been validated through execution in robotic platforms,[48,49] establishing that computationally generated routes can successfully guide experimental synthesis. The present work builds upon this established foundation but addresses a different research objective.

The primary contribution of this study lies in conceptual and algorithmic advances in how retrosynthetic reasoning is structured and explored computationally. In particular, the framework is designed to reflect how chemists initially reason about multistep synthesis at a strategic level for complex structures, while reformulating the problem in a way that improves the computational tractability of multistep search. Accordingly, the aim of this work is not to demonstrate the experimental realization of individual synthetic routes, but rather to establish that reformulating multistep retrosynthesis at a higher-level enables more effective ideation of retrosynthetic strategies, particularly for challenging targets.

Within this scope, experimental execution of a small number of representative routes, which necessarily involves subjective selection of targets and routes, would not meaningfully affect the validity of the central claims of this work. Successful experimental validation would provide anecdotal confirmation that a small number of specific pathways are chemically feasible but would not constitute a rigorous or systematic test of the general framework itself.

Further, the necessity of condition selection or screening introduces an additional degree of human intervention and subjectivity. Conversely, experimental challenges encountered in isolated cases would not invalidate the broader conceptual and computational advances introduced here. Given that experimental validation is most informative at downstream stages of synthesis planning, once strategic hypotheses have been refined to incorporate functional group compatibility and reaction conditions, and that experimental execution of one or a small number of pathways could neither substantiate nor refute the core contributions of this study, we consider it out of scope for the present work.

For these reasons, and consistent with the algorithmic nature of our contributions, we employ self-contained computational evaluation as the primary validation approach. We have focused on fully-reproducible computational benchmarks conducted across diverse targets, enabling transparent and objective comparisons of algorithm performance.

References

(1) Lowe, D. M. Chemical reactions from US patents (1976-Sep2016). 2017.

(2) The Pistachio dataset. `https://www.nextmovesoftware.com/pistachio.html`, accessed on 2025-03-01.

(3) Uwamori, M.; Osada, R.; Sugiyama, R.; Nagatani, K.; Nakada, M. In *Middle Molecular Strategy: Flow Synthesis to Functional Molecules*; Fukase, K., Doi, T., Eds.; Springer: Singapore, 2021; pp 111–126.

(4) Weininger, D. SMILES, a chemical language and information system. 1. Introduction to methodology and encoding rules. *Journal of Chemical Information and Computer Sciences* **1988**, *28*, 31–36.

(5) Coley, C. W.; Green, W. H.; Jensen, K. F. RDChiral: An RDKit Wrapper for Handling Stereochemistry in Retrosynthetic Template Extraction and Application. *Journal of Chemical Information and Modeling* **2019**, *59*, 2529–2537.

(6) NameRxn: Expert System for Named Reaction Identification and Classification. `https://www.nextmovesoftware.com/namerxn.html`.

(7) Chen, B.; Li, C.; Dai, H.; Song, L. Retro*: Learning Retrosynthetic Planning with Neural Guided A* Search. Proceedings of the 37th International Conference on Machine Learning. Online, 2020; pp 1608–1616.

(8) Mo, Y.; Guan, Y.; Verma, P.; Guo, J.; Fortunato, M. E.; Lu, Z.; Coley, C. W.; Jensen, K. F. Evaluating and clustering retrosynthesis pathways with learned strategy. *Chemical Science* **2021**, *12*, 1469–1478.

(9) Heid, E.; Liu, J.; Aude, A.; Green, W. H. Influence of Template Size, Canonicalization, and Exclusivity for Retrosynthesis and Reaction Prediction Applications. *Journal of Chemical Information and Modeling* **2022**, *62*, 16–26.

(10) Landrum, G. RDKit: Open-Source Cheminformatics Software. https://rdkit.org/.

(11) Biewald, L. Experiment Tracking with Weights and Biases. 2020; https://www.wandb.com/.

(12) Tetko, I. V.; Karpov, P.; Van Deursen, R.; Godin, G. State-of-the-art augmented NLP transformer models for direct and single-step retrosynthesis. *Nature Communications* **2020**, *11*, 5575.

(13) Tu, Z. et al. ASKCOS: Open-Source, Data-Driven Synthesis Planning. *Accounts of Chemical Research* **2025**, Publisher: American Chemical Society.

(14) Kocsis, L.; Szepesvári, C. Bandit Based Monte-Carlo Planning. Machine Learning: ECML 2006. Berlin, Heidelberg, 2006; pp 282–293.

(15) Daylight Chemical Information Systems, Inc., SMARTS - A Language for Describing Molecular Patterns. https://www.daylight.com/dayhtml/doc/theory/theory.smarts.html.

(16) Ertl, P.; Schuffenhauer, A. Estimation of synthetic accessibility score of drug-like molecules based on molecular complexity and fragment contributions. *Journal of Cheminformatics* **2009**, *1*, 8.

(17) Coley, C. W.; Rogers, L.; Green, W. H.; Jensen, K. F. SCScore: Synthetic Complexity Learned from a Reaction Corpus. *Journal of Chemical Information and Modeling* **2018**, *58*, 252–261.

(18) Böttcher, T. An Additive Definition of Molecular Complexity. *Journal of Chemical Information and Modeling* **2016**, *56*, 462–470.

(19) Krzyzanowski, A.; Pahl, A.; Grigalunas, M.; Waldmann, H. Spacial ScoreA Comprehensive Topological Indicator for Small-Molecule Complexity. *Journal of Medicinal Chemistry* **2023**, *66*, 12739–12750.

(20) Ertl, P.; Roggo, S.; Schuffenhauer, A. Natural Product-likeness Score and Its Application for Prioritization of Compound Libraries. *Journal of Chemical Information and Modeling* **2008**, *48*, 68–74.

(21) Bickerton, G. R.; Paolini, G. V.; Besnard, J.; Muresan, S.; Hopkins, A. L. Quantifying the chemical beauty of drugs. *Nature Chemistry* **2012**, *4*, 90–98.

(22) Liu, G.; Xue, D.; Xie, S.; Xia, Y.; Tripp, A.; Maziarz, K.; Segler, M.; Qin, T.; Zhang, Z.; Liu, T.-Y. Retrosynthetic Planning with Dual Value Networks. Proceedings of the 40th International Conference on Machine Learning. 2023; pp 22266–22276, ISSN: 2640-3498.

(23) Maziarz, K.; Tripp, A.; Liu, G.; Stanley, M.; Xie, S.; Gaiński, P.; Seidl, P.; Segler, M. H. S. Re-evaluating retrosynthesis algorithms with Syntheseus. *Faraday Discussions* **2025**, *256*, 568–586, Publisher: The Royal Society of Chemistry.

(24) Hassen, A. K.; Lai, H.; Genheden, S.; Preuss, M.; Clevert, D.-A. Synthesis Planning in Reaction Space: A Study on Success, Robustness and Diversity. 2025; https://chemrxiv.org/engage/chemrxiv/article-details/685d2f1a1a8f9bdab5e51a59.

(25) Schwaller, P.; Laino, T.; Gaudin, T.; Bolgar, P.; Hunter, C. A.; Bekas, C.; Lee, A. A. Molecular Transformer: A Model for Uncertainty-Calibrated Chemical Reaction Prediction. *ACS Central Science* **2019**, *5*, 1572–1583.

(26) Nicolaou, K. C.; Sorensen, E. J. *Classics in Total Synthesis: Targets, Strategies, Methods*; Wiley-VCH: New York, 1996.

(27) Draghici, C.; Njardarson, J. T. Chemistry By Design: A Web-Based Educational Flashcard for Exploring Synthetic Organic Chemistry. *Journal of Chemical Education* **2012**, *89*, 1080–1082.

(28) Lv, Z.; Song, C.; Niu, Y.; Li, Q.; Ye, X.-S. Synthesis of N-Substituted Iminosugar

Derivatives and Evaluation of Their Immunosuppressive Activities. *ChemMedChem* **2018**, *13*, 338–351.

(29) Fürstner, A.; Radkowski, K.; Wirtz, C.; Goddard, R.; Lehmann, C. W.; Mynott, R. Total Syntheses of the Phytotoxic Lactones Herbarumin I and II and a Synthesis-Based Solution of the Pinolidoxin Puzzle. *Journal of the American Chemical Society* **2002**, *124*, 7061–7069.

(30) Mitsunobu, O.; Yamada, M. Preparation of Esters of Carboxylic and Phosphoric Acid via Quaternary Phosphonium Salts. *Bulletin of the Chemical Society of Japan* **1967**, *40*, 2380–2382.

(31) Bansode, A. H.; Suryavanshi, G. Metal-free hypervalent iodine/TEMPO mediated oxidation of amines and mechanistic insight into the reaction pathways. *RSC Advances* **2018**, *8*, 32055–32062.

(32) McMurry, J. E.; Fleming, M. P. New method for the reductive coupling of carbonyls to olefins. Synthesis of .beta.-carotene. *Journal of the American Chemical Society* **1974**, *96*, 4708–4709.

(33) Sabelle, S.; Hydrio, J.; Leclerc, E.; Mioskowski, C.; Renard, P.-Y. McMurry intermolecular cross-coupling between an ester and a ketone: scope and limitations. *Tetrahedron Letters* **2002**, *43*, 3645–3648.

(34) Hirao, A.; Itsuno, S.; Nakahama, S.; Yamazaki, N. Asymmetric reduction of aromatic ketones with chiral alkoxy-amineborane complexes. *Journal of the Chemical Society, Chemical Communications* **1981**, 315–317.

(35) Davis, F. A.; Vishwakarma, L. C.; Billmers, J. G.; Finn, J. Synthesis of .alpha.-hydroxycarbonyl compounds (acyloins): direct oxidation of enolates using 2-sulfonyloxaziridines. *The Journal of Organic Chemistry* **1984**, *49*, 3241–3243.

(36) Dess, D. B.; Martin, J. C. Readily accessible 12-I-5 oxidant for the conversion of primary and secondary alcohols to aldehydes and ketones. *The Journal of Organic Chemistry* **1983**, *48*, 4155–4156.

(37) Kühnel, E.; Laffan, D. D. P.; Lloyd-Jones, G. C.; Martínez del Campo, T.; Shepperson, I. R.; Slaughter, J. L. Mechanism of Methyl Esterification of Carboxylic Acids by Trimethylsilyldiazomethane. *Angewandte Chemie International Edition* **2007**, *46*, 7075–7078.

(38) Yoo, J. W.; Seo, Y.; Park, J. B.; Kim, Y. G. Two-way homologation of aliphatic aldehydes: Both one-carbon shortening and lengthening via the same intermediate. *Tetrahedron* **2020**, *76*, 130883.

(39) Kieffer, M. E.; Repka, L. M.; Reisman, S. E. Enantioselective Synthesis of Tryptophan Derivatives by a Tandem Friedel–Crafts Conjugate Addition/Asymmetric Protonation Reaction. *Journal of the American Chemical Society* **2012**, *134*, 5131–5137.

(40) Nair, M. S. R.; Takeshita, H.; McMorris, T. C.; Anchel, M. Metabolites of Clitocybe illudens. IV. Illudalic acid, a sesquiterpenoid, and illudinine, a sesquiterpenoid alkaloid. *The Journal of Organic Chemistry* **1969**, *34*, 240–243.

(41) Morrison, A. E.; Hoang, T. T.; Birepinte, M.; Dudley, G. B. Synthesis of Illudinine from Dimedone. *Organic Letters* **2017**, *19*, 858–861.

(42) Chatare, V. K.; Andrade, R. B. Total Synthesis of ()-Albocycline. *Angewandte Chemie International Edition* **2017**, *56*, 5909–5911.

(43) Calvo-Peña, C.; Cobos, R.; Sánchez-López, J. M.; Ibañez, A.; Coque, J. J. R. Albocycline Is the Main Bioactive Antifungal Compound Produced by Streptomyces sp. OR6 against Verticillium dahliae. *Plants* **2023**, *12*, 3612.

(44) Helquist, P.; Bergdahl, M.; Hett, R.; Gangloff, A. R.; Demillequand, M.; Cottard, M.; Mader, M. M.; Friebe, T.; Iqbal, J.; Wu, Y.; Akermark, B.; Rein, T.; Kann, N. Synthesis of macrocyclic lactam/lactone derivatives having antimicrobial activity. *Pure and Applied Chemistry* **1994**, *66*, 2063–2066, Publisher: De Gruyter.

(45) Li, Q.; Seiple, I. B. Modular, Scalable Synthesis of Group A Streptogramin Antibiotics. *Journal of the American Chemical Society* **2017**, *139*, 13304–13307, Publisher: American Chemical Society.

(46) Klucznik, T. et al. Efficient Syntheses of Diverse, Medicinally Relevant Targets Planned by Computer and Executed in the Laboratory. *Chem* **2018**, *4*, 522–532.

(47) Mikulak-Klucznik, B. et al. Computational planning of the synthesis of complex natural products. *Nature* **2020**, *588*, 83–88, Publisher: Nature Publishing Group.

(48) Coley, C. W. et al. A robotic platform for flow synthesis of organic compounds informed by AI planning. *Science* **2019**, *365*, eaax1566.

(49) Koscher, B. A. et al. Autonomous, multiproperty-driven molecular discovery: From predictions to measurements and back. *Science* **2023**, *382*, eadi1407, Publisher: American Association for the Advancement of Science.
